# Technical versus biological variability in a synthetic human gut community

**DOI:** 10.1080/19490976.2022.2155019

**Published:** 2022-12-29

**Authors:** Charlotte van de Velde, Clémence Joseph, Kenneth Simoens, Jeroen Raes, Kristel Bernaerts, Karoline Faust

**Affiliations:** a KU Leuven, Department of Microbiology, Immunology and Transplantation, Rega Institute for Medical Research, Laboratory of Molecular Bacteriology, Leuven B-3000, Belgium; b KU Leuven, Department of Chemical Engineering, Chemical and Biochemical Reactor Engineering and Safety (CREaS), Leuven B-3001, Belgium; c Center for Microbiology, VIB-KU Leuven, Leuven, Belgium

**Keywords:** microbioreactor, human gut microbiota, Blautia, Bacteroides, synthetic community, chemostat

## Abstract

Synthetic communities grown in well-controlled conditions are an important tool to decipher the mechanisms driving community dynamics. However, replicate time series of synthetic human gut communities in chemostats are rare, and it is thus still an open question to what extent stochasticity impacts gut community dynamics. Here, we address this question with a synthetic human gut bacterial community using an automated fermentation system that allows for a larger number of biological replicates. We collected six biological replicates for a community initially consisting of five common gut bacterial species that fill different metabolic niches. After an initial 12 hours in batch mode, we switched to chemostat mode and observed the community to stabilize after 2–3 days. Community profiling with 16S rRNA resulted in high variability across replicate vessels and high technical variability, while the variability across replicates was significantly lower for flow cytometric data. Both techniques agree on the decrease in the abundance of *Bacteroides thetaiotaomicron*, accompanied by an initial increase in *Blautia hydrogenotrophica*. These changes occurred together with reproducible metabolic shifts, namely a fast depletion of glucose and trehalose concentration in batch followed by a decrease in formic acid and pyruvic acid concentrations within the first 12 hours after the switch to chemostat mode. In conclusion, the observed variability in the synthetic bacterial human gut community, as assessed with 16S rRNA gene sequencing, is largely due to technical variability. The low variability seen in HPLC and flow cytometry data suggests a highly deterministic system.

## Introduction

The human gut microbiome is a complex and dynamic ecosystem, containing numerous microbes that perform different functions. They produce essential nutrients such as vitamins and short-chain fatty acids (SCFA).^1,2^ In addition, the human gut microbiome plays an important role in the regulation of the host immune system, which is essential for health.^3^

Longitudinal cohort studies show that the composition of the human gut microbiome as assessed with fecal samples does not change substantially when unperturbed,^4,5^ but that day-to-day variation is nevertheless visible. This poses the question whether short-term variation is due to small changes in the environment (e.g. diet or circadian rhythms of the host),^6,7^ the intrinsic stochasticity of the system (e.g. variable growth and death rates), or technical variability.

Here, our goal is to investigate the reproducibility of the dynamics of a defined human gut bacterial community to quantify the variability in gut microbial abundances in well-controlled conditions. To do this, we need to work with a single source of inoculum and control all external sources that influence microbial dynamics. The latter is only possible in a bioreactor where atmosphere, temperature, and pH are kept constant, and the process can be operated in chemostat conditions in which a steady state can be reached. This requires a continuous culture to regularly add fresh nutrients and remove waste products. In such well-controlled conditions, the remaining variation is either due to community dynamics (i.e. stochasticity, but also nearby alternative stable states or chaos^8^), short-term evolution (not explored here), or technical variability of measurements. Although variability of microbial gut community dynamics can also be assessed with fecal slurries,^9,10^ working with a defined community makes it easier to understand the mechanisms driving the dynamics as it is less complex than fecal slurries, lacking archaea, fungi, and viruses.

Bioreactor systems range in complexity from coupled vessels mimicking the human intestinal system to well plates,^11,12,33^ representing a trade-off in realism and control versus the number of replicates that can be achieved. Although well plates combined with serial transfer are claimed to emulate chemostat conditions, they instead represent a sequential batch process (i.e., a series of (short) batch experiments). Traditional chemostats in bench-top bioreactors adopt a continuous inflow of fresh medium and outflow of broth keeping the volume constant. A series of such chemostats (with different specific process parameters) have been coupled to mimic the intestinal tract.^11^ Since such bench-top chemostat bioreactors are cost-, time- and labor-intensive, replicate time series of defined human gut microbial communities in such chemostats are rare. The few that have been collected show that gut communities reach a stable state within a few days and that the dynamics are reproducible, but that there is some variability across biological replicates.^14–16^ However, the number of replicates in these experiments is small and cell densities are usually not measured, such that conclusions rely on relative instead of absolute abundances.

Here, we monitored microbial community composition in six parallel bioreactors in chemostat mode to investigate the variability of the dynamics of a defined human gut community and obtained total cell counts via flow cytometry. The community was composed of gut bacterial species with different metabolic roles, including the butyrate producer *Roseburia intestinalis* DSM 14610 (RI),^17^ the acetogen *Blautia hydrogenotrophica* DSM 10507 (BH),^18,19^ the lactate producer *Collinsella aerofaciens* RCC 1377 (CA),^20^ and the succinate producers *Bacteroides thetaiotaomicron* DSM 2079 (BT)^21^ and *Prevotella copri* DSM18205 (PC).^22^ The two latter species are also representatives of two different enterotypes.^23,24^ We calculated the total number of cells in each sample using flow cytometry and systematically assessed the technical variability of 16S rRNA gene sequencing. As an alternative to the latter, we also applied supervised classification to flow cytometry data to classify events by species. This method has already been validated in the context of gut bacteria, where a synthetic community comprised four bacteria (RI, BH, BT, and FP) was evaluated.^25^ In both cases, we observed a reproducible change in composition before the community stabilized.

## Results

### Transient community dynamics is characterized by reproducible metabolic changes

**Figure 1.**
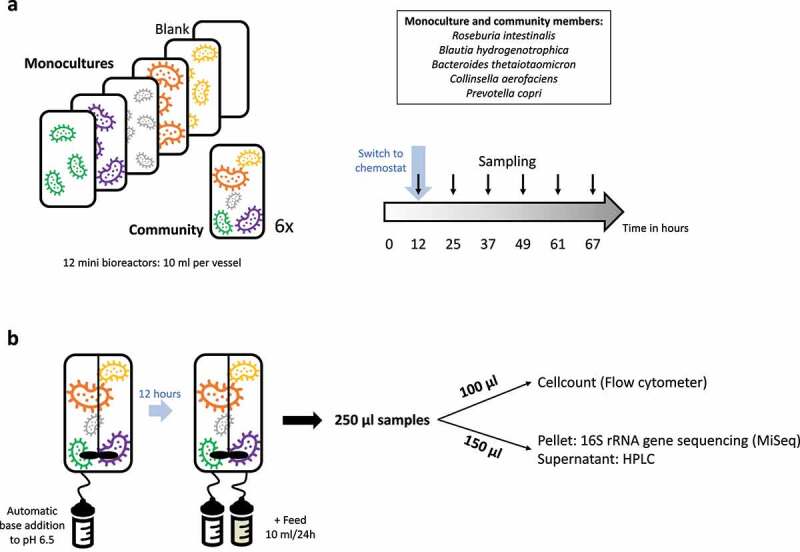
Experimental setup. a) Twelve microbioreactor vessels are inoculated with five monocultures (*Roseburia intestinalis, Blautia hydrogenotrophica, Bacteroides thetaiotaomicron, Collinsella aerofaciens* and *Prevotella copri*) and six replicates for the synthetic community. However, *Prevotella* was not consistently detected in the communities after the batch phase. b) After an initial twelve hours in batch where the pH was kept constant at pH 6.5, the system was switched to chemostat mode. Samples were taken at specific timepoints and subsequently processed for cell count, 16S rRNA gene sequencing and HPLC.

Monocultures and communities of five gut bacterial species were inoculated in Wilkins-Chalgren medium, and community composition was followed for 67 hours in six biological replicates. All microbioreactors were inoculated from the same pool of cells for each experiment, respectively, with the same number of cells/ml for each species as determined by flow cytometry. We started the chemostat mode after 12 hours of batch incubation, to allow the slower-growing species to reach sufficiently high densities to avoid wash-out (e.g. BH and PC, see Supplementary Figure 1). Since the system has no automatic outflow of medium, we set up a pipetting scheme where excess liquid was pipetted out every 2 hours during the day and every 4 hours during the night, thus creating an emulated chemostat. An overview of the experimental setup is given in Figure 1.

**Figure 2.**
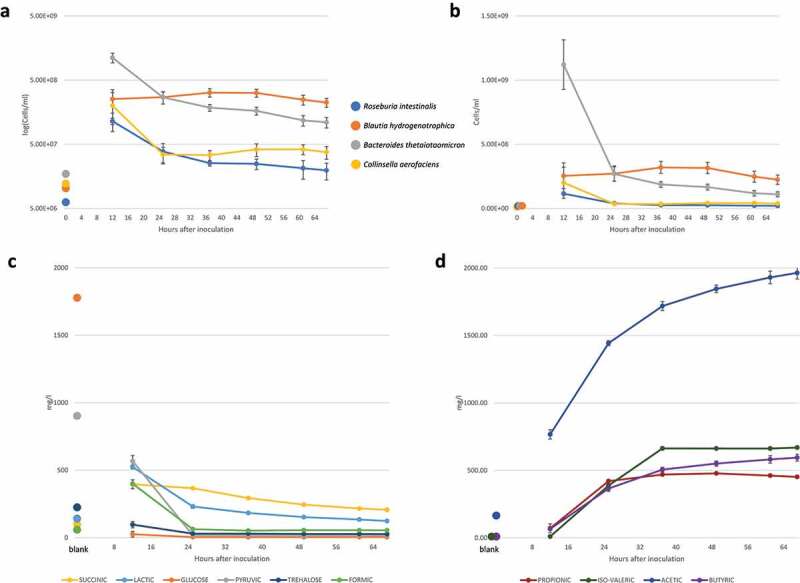
Microbial abundances assessed with 16S rRNA gene sequencing (corrected for copy number) and metabolite concentrations over time for the synthetic gut community. a) The mean and standard deviation of species abundance across six biological replicates is shown in logarithmic scale. For timepoint ‘0’, flow cytometry data of the monocultures for the inoculum was used. b) The same as a, without the logarithmic scale. a,) Only species consistently present across technical replicates and time points are shown. See Supplementary Figure 3 for time series obtained with the second and third technical replicate. c) Mean concentrations for metabolites with decreasing concentrations across the six replicates. d) Mean concentrations for metabolites with increasing concentrations across the six replicates. For the metabolite data, timepoint 0 represents the blank medium. For all graphs, error bars represent standard deviation across the biological replicates.

We combined 16S rRNA gene sequencing with flow cytometry to measure absolute species abundances at specific timepoints (Figure 2 a and b). PC declined quickly and was on the border of the detection limit, but the abundances of the other four species stabilized at the end of the experiment (i.e., 2–3 residence times). We also performed a validation experiment with a modified community composition (*Faecalibacterium prausnitzii* instead of CA), which also stabilized (Supplementary Figure 2). The abundance of BT decreased over time, while BH increased initially. The dominance of BT in the first 12 hours is expected since it grows faster than the other strains in the chosen medium (Supplementary Figure 1).

**Figure 3.**
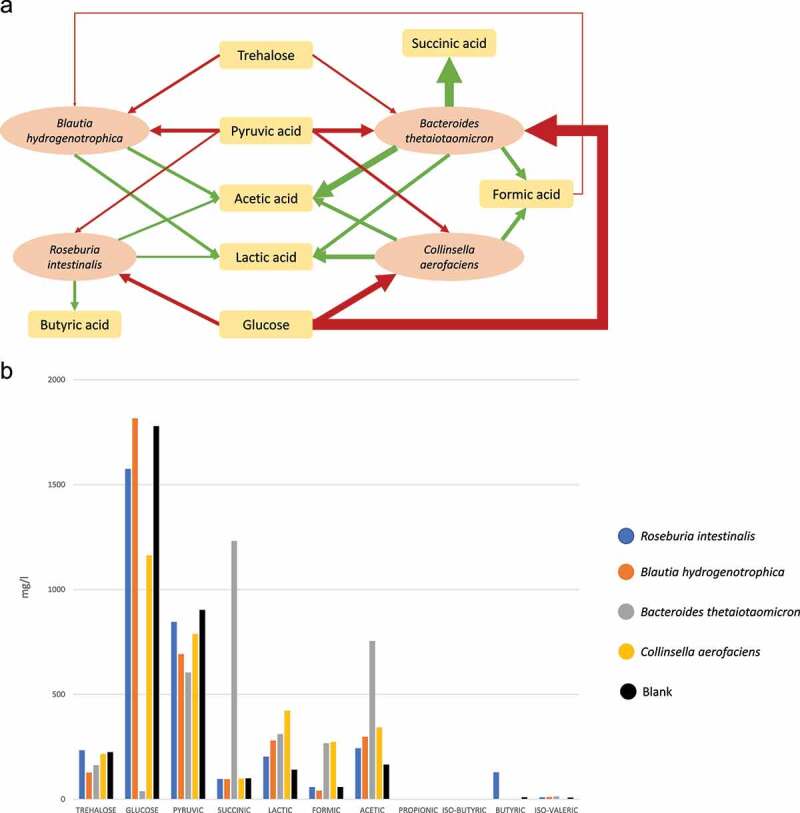
a) Schematic representation of produced (green) and consumed (red) metabolites at T = 12 hours after inoculation in batch fermentation in monoculture. The thickness of the arrow indicates the relative amount of production/consumption compared to the blank (see b). b) HPLC results for monocultures after 12 h in batch mode. *Prevotella copri* was excluded due to contamination (see Supplementary Figure 7). The arrow thickness was scaled by taking ½ of the square root.

Next, we looked at the metabolite concentrations (Figure 2 c and d). Over time, acetic, butyric, propionic, and iso-valeric acid concentrations increased, while trehalose, glucose, pyruvic, succinic, lactic, and formic acid decreased. The transient dynamics is characterized by two phases. First, we saw a net increase in formic, succinic, and lactic acid, a depletion in glucose and pyruvic acid, and a decrease in trehalose in the first 12–25 hours. Subsequently, succinic, lactic, and formic acid decreased before metabolites stabilized. This decrease could be either due to consumption or to reduced production. This dynamic is reproduced in the six biological replicates for which metabolites were measured. Metabolite measurements of monocultures after 12 hours of incubation show that BH did not consume glucose, while BT and to a lesser extent RI and CA consumed it (Figure 3, Supplementary Figure 4) suggesting that BT was the main driver of its depletion at 12 hours in the community. In addition, no organism consumed lactate in the monoculture during the batch phase, but it decreased in the transient phase of the chemostat. Succinic acid was first produced and decreased after 25 hours. It has no consumer according to the monocultures in batch but is significantly correlated with *Bacteroides*, its only producer in monoculture (Supplementary Figure 5). Butyric acid, acetic acid, and iso-valeric acid, which are produced in the monocultures, increase in the community until reaching steady state.

### Technical variability of 16S rRNA gene sequencing exceeds biological variability

**Figure 4.**
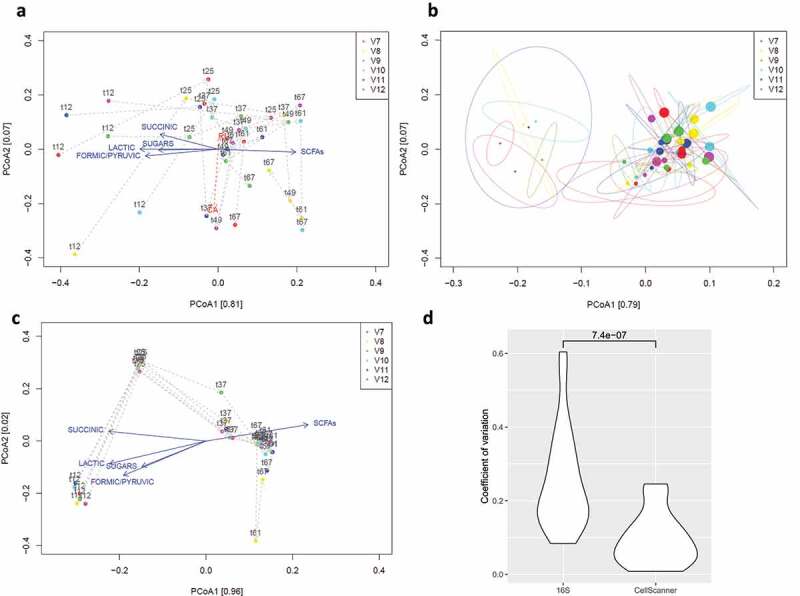
PCoA on relative abundances from 16S rRNA gene sequencing (corrected for copy number) and flow cytometry data processed with CellScanner. Metabolites significantly correlated to principal components were added using envfit. a) First technical replicate of 16S rRNA gene sequencing for six biological replicates and six timepoints. b) 75% confidence areas calculated on three technical replicates of 16S rRNA gene sequencing for six biological replicates and six time points. Dot size encodes time. c) CellScanner results for six biological replicates and six timepoints. a-c) Color encodes biological replicate. d) Distributions of CV (coefficient of variation) values of relative abundances per species and time point across vessels. The CV values of the first technical replicate are significantly higher than for CellScanner (Wilcoxon signed-rank test).

To explore the sources of variability in the data, we assessed the technical variability of 16S rRNA gene sequencing. For this, we extracted, amplified, and sequenced DNA from all biological replicates for each time point three times. The two replicates that were processed together were more similar to each other than either was to the third replicate (Supplementary Figure 6). The three technical replicates reproduced the main trend (decrease of BT and increase of BH, Supplementary Figure 3). However, the technical variability of 16S rRNA gene sequencing is larger than the variability between the replicate vessels (Figure 4b, Supplementary Figures 8, 9). We calculated the coefficient of variation (CV) for the results of HPLC, flow cytometry (CellScanner, see below), and 16S rRNA gene sequencing (Tables 1–4) and found that the CV of 16S rRNA relative abundances are significantly higher than for those obtained with CellScanner (Figure 4d, paired Wilcoxon signed rank test p-value < 0.001).

**Table 1.** Coefficient of variation across vessels for metabolite data

Time in hours	SUCROSE	GLUCOSE	PYRUVIC	SUCCINIC	LACTIC	FORMIC	ACETIC	PROPIONIC	BUTYRIC	ISO-VALERIC	Average per timepoint
12	0.244388	0.858503	0.072397	0.083457	0.02851	0.021789	0.044231	0.463254	0.148036	0.440977	0.240554143
25	0.017016	0.145383	0.005845	0.024062	0.056295	0.012329	0.012509	0.011448	0.056756	0.10848	0.045012224
37	0.015275	0.075146	0.026605	0.036005	0.033019	0.043567	0.01939	0.013149	0.034249	0.016311	0.031271589
49	0.006412	0.081673	0.030288	0.028576	0.038954	0.038535	0.015391	0.006143	0.035086	0.00906	0.02901184
61	0.008326	0.15639	0.023726	0.037439	0.044986	0.08773	0.023782	0.011166	0.048077	0.01169	0.045331084
67	0.027965	0.128629	0.031992	0.051787	0.044778	0.081846	0.023155	0.015654	0.043894	0.014131	0.046382998
Average per metabolite	0.053231	0.240954	0.031809	0.043554	0.04109	0.047633	0.023076	0.086802	0.061016	0.100108

**Table 2.** Coefficient of variation across vessels for absolute and relative strain abundances obtained with CellScanner

Time in hours	*Roseburia intestinalis*	*Blautia hydrogenotrophica*	*Bacteroides thetaiotaomicron*	*Collinsella aerofaciens*	unknown	Average per timepoint
**CellScanner Coefficient of variation ABS**						
12	0.083778	0.035507	0.023773	0.087737	0.128904	0.07194
25	0.265497	0.187063	0.190103	0.206035	0.171997	0.204139
37	0.188714	0.108122	0.156754	0.180169	0.142749	0.155302
49	0.146688	0.129058	0.182511	0.217926	0.145821	0.164401
61	0.307789	0.138084	0.234025	0.173425	0.193095	0.209284
67	0.272897	0.152637	0.247013	0.18302	0.170893	0.205292
Average per strain	0.210894	0.125079	0.172363	0.174719	0.15891	
**CellScanner Coefficient of variation REL**						
12	0.08524	0.036112	0.022186	0.088633	0.130591	0.072552
25	0.214333	0.010937	0.007273	0.046363	0.030389	0.061859
37	0.214488	0.028629	0.053429	0.077235	0.029701	0.080696
49	0.073805	0.01982	0.057038	0.117559	0.051704	0.063985
61	0.169425	0.034899	0.128028	0.224111	0.053825	0.122058
67	0.139789	0.056921	0.107093	0.103975	0.024723	0.0865
Average per strain	0.149513	0.03122	0.062508	0.109646	0.053489

**Table 3.** Coefficient of variation across vessels for the first technical replicate of 16S rRNA gene sequencing. Pseudomonas is a contaminant detected in a sub-set of the samples in low amounts.

Coefficient of variation REL 1st 16S technical replicate												
Time in hours	*Roseburia intestinalis*	*Blautia hydrogenotrophica*	*Bacteroides thetaiotaomicron*	*Collinsella aerofaciens*	*Prevotella copri*	Pseudomonas	Average per timepoint	Average per timepoint without Prevotella/Pseudomonas				
12	0.15564025	0.342347337	0.084569975	0.444253232	1.253757964	2.236067977	0.752772789	0.256702699				
25	0.245641378	0.12732518	0.072919997	0.229917778	1.00863528	1.42241808	0.517809616	0.168951083				
27	0.142818618	0.045539634	0.032834755	0.226996463	2.236067977	2.236067977	0.820054237	0.112047367				
49	0.141334216	0.026952911	0.040465197	0.126509944	2.236067977	1.506706746	0.679672832	0.083815567				
61	0.184331535	0.016473304	0.01774408	0.175607974			0.098539223	0.098539223				
67	0.193706912	0.032947196	0.036114669	0.094963831		1.520979059	0.375742333	0.089433152				
Average per strain	0.177245485	0.098597594	0.047441445	0.21637487	1.6836323	1.784447968						
Coefficient of variation ABS 1st 16S technical replicate												
Time in hours	*Roseburia intestinalis*	*Blautia hydrogenotrophica*	*Bacteroides thetaiotaomicron*	*Collinsella aerofaciens*	*Prevotella copri*	Pseudomonas	Average per timepoint	Average per timepoint without Prevotella/Pseudomonas				
12	0.113239276	0.397144213	0.172832451	0.60641672	1.242717343	2.236067977	0.79473633	0.322408165				
25	0.33068139	0.22071944	0.210248005	0.287588766	1.003694528	1.415466593	0.578066454	0.2623094				
27	0.102026264	0.146570623	0.120770959	0.172268032	2.236067977	2.236067977	0.835628639	0.13540897				
49	0.186705056	0.140052997	0.138907605	0.222581161	2.236067977	1.530790053	0.742517475	0.172061705				
61	0.324258986	0.176068622	0.187582764	0.176863057			0.216193357	0.216193357				
67	0.292911099	0.162137207	0.184128334	0.226700163		1.447941079	0.462763576	0.216469201				
Average per strain	0.224970345	0.207115517	0.169078353	0.28206965	1.679636957	1.773266736

**Table 4.** Coefficient of variation across vessels and strains.

Timepoint	HPLC	CellScanner (relative abundance)	CellScanner (absolute abundance)	16S 1 (relative abundance)	16S 1 (absolute abundance)	16S 2 (relative abundance)	16S 2 (absolute abundance)	16S 3 (relative abundance)	16S 3 (absolute abundance)
12	0.24	0.07	0.07	0.26	0.32	0.22	0.33	0.24	0.23
25	0.05	0.06	0.20	0.17	0.26	0.21	0.29	0.20	0.31
37	0.03	0.08	0.16	0.11	0.14	0.20	0.21	0.26	0.27
49	0.03	0.06	0.16	0.08	0.17	0.27	0.32	0.26	0.31
61	0.05	0.12	0.21	0.10	0.22	0.24	0.32	0.18	0.25
67	0.05	0.09	0.21	0.09	0.22	0.23	0.32	0.27	0.33

### Abundances obtained through flow cytometry data analysis reproduce the main trend

**Figure 5.**
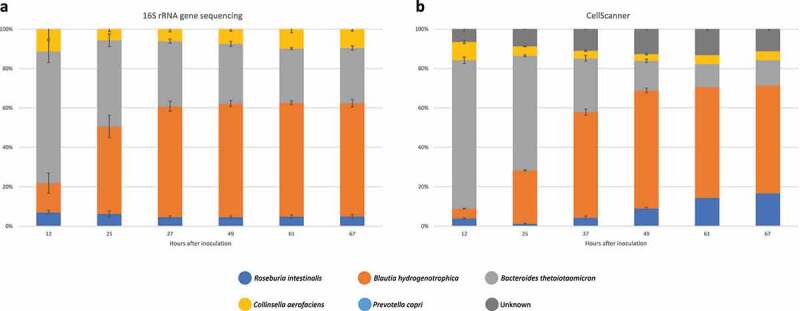
Comparison of 16S rRNA gene sequencing results (corrected for copy number) and CellScanner. a) Mean relative abundance of gut bacterial species by 16S rRNA gene sequencing across six biological replicates for the first technical replicate. b) Mean relative abundance of gut bacterial species by CellScanner across six biological replicates (see Supplementary Figure 10C for the CellScanner proportions without unknowns). Events on which the classifiers did not agree are classified as unknown. Error bars represent standard deviation.

Given the high technical variability of 16S rRNA gene sequencing, we used an alternative method to detect and quantify bacterial species over time. CellScanner trains classifiers (random forests) on monoculture flow cytometry data, which then determines the species of an event in a community flow cytometry file. CellScanner also performs automated gating on monocultures and blanks using the same principle. To assess how well different supervised classification methods in CellScanner can distinguish the species, we evaluated them on communities assembled in silico from mono-culture data (Supplementary Figure 10A and 10B). CellScanner agrees with 16S rRNA gene sequencing on the same trend: the abundance of BT decreased and that of BH increased, while RI and CA remain relatively constant (Figure 5). More precisely, for BH and BT, CellScanner and sequencing abundances are significantly correlated. For CA, they are weakly correlated, and for RI, the techniques disagree (Supplementary Table S1). In agreement with the lower CV values seen for CellScanner, PCoA plots show that the trajectories of individual vessels are closer to each other when assessed with flow cytometry data as opposed to sequencing data (Figure 4 a and c).

### Dynamics of total cell counts and heterogeneity

**Figure 6.**
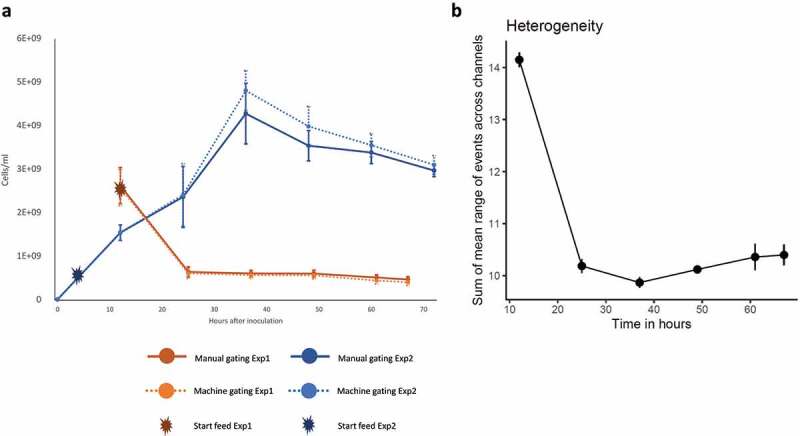
a) The cell density decreases significantly over time for the first data set (exp1, Pearson’s r: −0.76). The error bar shows the standard deviation across six biological replicates for the first experiment and three biological replicates for the validation experiment (exp2). Two different gating techniques were applied to distinguish cells from debris in flow cytometry data, namely manual gating and gating through supervised classification as implemented in CellScanner. The full graph of the validation experiment is shown in Supplementary Figure 11. b) Heterogeneity of flow cytometry data shown across the six biological replicates for the first data set.

There was a reproducible decline in total cell counts between the end of the batch phase at 12 h and 25 h (Figure 6a, Wilcoxon rank-sum test p-value 0.0022). In the validation experiment, where the initial batch phase was reduced to 4 hours, we see a decline in cell count after 36 hours. In both experiments, the decline in total cell count is accompanied by a decrease in the abundance of BT (Figure 2 a and b, Supplementary Figure 2A-B), the most abundant species at both time points. Taken together, this suggests that the decline in the BT population is responsible for the strong initial decline in cell numbers. Next, we quantified population heterogeneity as the mean of the range of flow cytometry channel values (e.g. the values of each of the 23 flow cytometry parameters, see Material and methods) and observed a large change within the first 24 hours, perhaps due to dead cells that are washed out in chemostat mode. Intriguingly, the heterogeneity then increases again over time (Figure 6b), though the difference between 25 and 67 hours is not significant (Wilcoxon rank-sum test p-value 0.09).

## Discussion

Here, we investigated the dynamics of a defined community of human gut bacteria in continuous culture in a comparatively large number of replicates, while maintaining a high degree of environmental control. To our knowledge, this is the first time that the dynamics of a synthetic human gut bacterial community is investigated in chemostat mode using an automated multi-bioreactor system.

A limitation of the current study is the frequent contamination in monocultures despite their location inside a HEPA-filtered anaerobic chamber. We adopted a stricter cleaning routine for the validation experiment, which reduced contamination in the community below the detection level. Another limitation is that the growth stage of the inoculated strains (18 h pre-cultures) may affect the dynamics of the community, which was not further explored here.

We found that species abundances and metabolite concentrations in the community were reproducible across replicates, in agreement with previous results.^14,16^ However, we also observed that the technical variability of 16S rRNA gene sequencing exceeded the variability across vessels. Since we repeated the entire sequencing protocol three times, we do not know whether variation comes from DNA extraction, PCR, or the sequencing runs. We positioned blanks randomly and differently across replicate well plates, to avoid bias due to well-to-well contamination. Previous studies using mock communities have shown that most of the technical variation is due to extraction and amplification and not from the sequencing step itself.^26,27^

The high technical variability of 16S rRNA gene sequencing limits how much biological variability we can see. Therefore, we also applied an alternative method for the analysis of community composition based on flow cytometry data, implemented in CellScanner. Here, we found that trajectories across biological replicates were more similar to each other than they were for 16S rRNA sequencing data. Together with the low variation of metabolite data, this suggests that the technical variability of 16S rRNA sequencing inflates the variability observed with sequencing data and that the true biological variability is lower. However, CellScanner results are also potentially biased, since only the first monoculture time point was usable for BT and CA because of subsequent contamination. Changes in cell structure due to physiological changes from batch to continuous mode or species interactions could not be considered when training the classifiers. Thus, neither 16S rRNA gene sequencing nor CellScanner may have captured the exact composition. Alternative techniques such as whole-genome shotgun and full-length 16S rRNA gene sequencing are worth mentioning in this context, but they are work-intensive and it is questionable whether they reduce the technical variability compared to 16S rRNA gene sequencing. Quantitative PCR with species-specific primers is another alternative that delivers absolute abundances, which were observed previously to agree in sum with flow cytometry counts.^33^ The advantage of supervised classification of flow cytometry data is that it gives absolute abundances without DNA extraction. However, like species-resolved quantitative PCR, it does not scale to complex communities.^25^ Obtaining reliable species-specific cell counts in a high-throughput manner remains a major challenge when exploring community dynamics.

Both CellScanner and 16S rRNA gene sequencing agree on the main trend, namely a decrease in BT and an increase in other species, notably BH. BT grows faster than BH in batch. In chemostat, BT cannot sustain its high growth rate given the glucose feed and its abundance therefore declines. Other species are less dependent on glucose and exploit alternative carbon sources such as pyruvate. This is especially true for BH, which did not consume glucose in the first 12 h but lowered the pyruvate and trehalose concentrations instead. Interestingly, in an unintended invasion experiment of BH versus BT (initially, a BT monoculture), BH dominated at 67 h (Supplementary Figure 7A). However, BT won over BH in a two-species co-culture where a chemostat was emulated through serial transfer.^13^ This may be due to the fact that the medium in the serial-transfer experiment contained starch in addition to sugar, which may have given BT, a known carbohydrate specialist,^28,29^ an edge. In our experiments, BT displays the behavior of a pioneer species that quickly exploits an easily accessible carbon source but is then replaced, to an extent, by slower growing but metabolically more versatile species. This also fits observations in cohort studies, which show that the *Bacteroides* 2 enterotype dominates infant microbiota after weaning^30^ but is less frequent in adults. PC disappeared quickly from the community; after 25 hours the abundance was already bordering the detection limit of 16S rRNA gene sequencing. We do not know whether it was surviving in the community with a cell density below the detection limit, whether it was washed out, or whether it was outcompeted by other species in the community. A serial transfer experiment in a similar medium previously showed that BT outcompetes PC.^13^

Transient dynamics in which a species loses its dominant position was also observed in two other continuous cultures with defined human gut communities. In both cases, the community was first dominated by *Escherichia coli*, which then declined in favor of *Bacteroides thetaiotaomicron*.^14,15^ The observations described here are to our knowledge the first instance of a defined human gut community where a *Bacteroides* species decreases while other gut species increase and as such may contribute to understanding the enterotype shifts observed in fecal time-series data.

The metabolite consumption and production patterns agree with previous observations and with predictions based on metabolic reconstructions from the AGORA database (Supplementary Table S2).^31^ For instance, we saw a decrease in formate in the community. It has been shown before that formate produced in co-culture can be converted to acetate by BH using the Wood-Ljungdahl pathway.^32–34^ It is a challenge to disentangle the contributions of different species to metabolite concentrations in a community. Although meta-omics can provide clues of pathway activation in a community context in different conditions,^35^ it does not measure metabolite uptake and production rates. We saw that some species-metabolite correlations computed from co-culture data agree with monoculture data (e.g. BH consumes pyruvate, trehalose, and formate and produces acetate in monoculture and is negatively correlated with the three former and positively with the latter for CellScanner data) but there are also several correlations that do not reflect consumption/production (such as the negative correlation of BH with succinate, which is an indirect relationship due to the decrease of BT, the only succinate producer). Species may alter their metabolism when encountering low levels of their preferred nutrient and/or in the presence of interaction partners,^32^ and thus determining who produces and consumes what in this community is a task for the future.

Our data suggest that the community reached a steady state. We also note that all replicates followed similar trajectories i.e. only one state was reached. It is possible that this community does not have alternative stable states (i.e. it is not multi-stable). Alternatively, the different stable states for the species combination could be too far apart for small variations to trigger a change in community state. A different community state might also be too rare to see using six replicates. Further research is necessary to test whether initial differences in species abundances or perturbations would alter the final state reached.

In conclusion, we have shown that the defined human gut community investigated here is a highly deterministic system where transient dynamics (succession) is reproducible, and the observed variation is due in large part to the technical variability of 16S rRNA gene sequencing data. This further encourages the development of mathematical models for gut microbial communities.

## Material and methods

### Strains and inoculum preparation

The following gut bacterial species were used in this study: *Roseburia intestinalis* (DSM 14610), *Blautia hydrogenotrophica* (DSM 10507), *Bacteroides thetaiotaomicron* (DSM 2079), *Faecalibacterium prausnitzii* (DSM 17677), *Prevotella copri* (DSM 18205), and *Collinsella aerofaciens* (Raes Culture Collection 1366).

All bacteria were precultured twice at 37°C without agitation under anaerobic conditions in a Don Whitley A135 or A35 Anaerobic Workstation with HEPA filter (10% H_2_, 10% CO_2_, 80% N_2_, 55% humidity). First, strains were precultured for 48 hours in a modified Gifu Anaerobic Medium broth (mGAM,^36^ HyServe), except for *F. prausnitzii* DSM17677, which was grown in Reinforced Clostridial Medium broth (RCM, Oxoid). The strains were subsequently cultured for 18 hours in Wilkins-Chalgren anaerobe broth (WC, Oxoid) before inoculation in the Ambr bioreactors.

The microbioreactors were inoculated to a concentration of roughly 1*10^6^ – 8*10^6^ cells per ml. The number of cells for this inoculation was determined by flow cytometry, after which the cells were diluted in a WC medium and in the case of the community cultures mixed together in even ratios. 1 ml of each monoculture or community inoculum was used to inoculate the bioreactors to a total volume of 10 ml. Continuous feeding and sampling started 12 hours after inoculation to prevent washout of slower-growing strains. Emulated chemostat fermentations were performed using an Ambr® 15 Fermentation (Ambr 15 f) system (Sartorius Stedim Biotech, Royston, UK) located inside a Don Whitley A155 Anaerobic Workstation with a HEPA filter (10% H_2_, 10% CO_2_, 80% N_2_, 55% humidity). We used one culture station containing 12 single-use microbioreactors (Figure 1a) with a working volume of 8–12 ml. The vessels were kept at 37°C (± 0.3°C) by individual vessel heaters, while each vessel was stirred at 300 rpm by a single Rushton-like impeller. Each vessel contains optical sensors for pH and dissolved oxygen (DO) monitoring (12 s per cycle).

The Ambr 15 f has a liquid handler for automated pipetting and two pumped liquid lines to each individual vessel for feed and base addition that deliver liquid in 5 μl shots. The feed (WC anaerobe broth) was pumped in continuously at 6.9 μl/min, resulting in a complete changeover of the medium in the vessels in 24 hours. Samples (250 μl) were pipetted into a cooled plate (4°C). To prevent the vessels from overflowing due to the continuous feed, and to create an emulated chemostat, excess liquid was pipetted out at regular intervals into a waste plate to maintain an approximate volume of 10 ml per bioreactor. During daytime, 840 μl was pipetted out every 2 hours. During the night, 1680 μl was pipetted out every 4 hours.

The pH was measured online using fluorescent sensor patches, and an additional base (0.5 mM NaOH) was automatically added with a target pH of 6.5 and a trigger pH of 6.3 if needed. The pH was measured offline using an analysis module, which automatically re-calibrates the online sensors if necessary. A minimum of 0.3 ml (blank) up to 1.6 ml (BT monoculture) of base was added over a period of 67 hours. Dissolved oxygen tension (DOT) was measured online using fluorescent sensor patches, but as we keep the Ambr 15 f in a controlled, oxygen-free environment, it was measured below 0.25% during all experiments. While this experimental design enabled parallel fermentation and automatization of sample collection, it did not allow quantifying the concentrations of gases.

One hundred microliters of the samples taken were used for cell count by flow cytometry. The rest was split in half in 96-well plates (75 μl each), and the cells were pelleted by centrifugation at 3220 RCF for 10 minutes. Both pellet- and supernatant plates were stored at −80°C for further use. Samples taken at 12, 25, 37, 49, 61 and 67 hours after inoculation were used for16S rRNA gene sequencing (pellets) and HPLC (supernatant).

### Validation experiment

The inoculum for the validation experiment was based on OD600, where we inoculated RI, BH, and FP with a final OD of 0.004 and BT with a final OD of 0.002 in triplicate. The pH was monitored but not adjusted. We started the feed earlier at 4 hours after the inoculation. Excess medium was taken out every 3 hours after starting the feed, including samples every 6 hours. In addition, we ran this experiment for 258 hours.

### Cell counting with flow cytometry

For the inoculation, monocultures were serially diluted 1000x in PBS to an approximate cell density of 10^6^ cells per ml. The samples taken during the experiment were serially diluted 100x in PBS. All the samples were stained with 1 μl/ml SYBR green I (1:100 dilution in dimethyl sulfoxide; 20 min incubation at 37°C; 10.000 concentrate, Thermo Fisher Scientific) following the protocols previously described.^25,32,37–39^

A CytoFLEX S flow cytometer (Beckman Coulter) was used in the experiments. This resulted in a multiparametric description of each sample consisting of 23 parameters (FSC-A, FSC-H, SSC-A, SSC-H, FL1-A, FL1-H, FL2-orange-A, FL2-orange-H, FL3-red-A, FL3-red-H, FL4-A, FL4-H, APC-A750-A, APC-A750H, VSSC-A, VSSC-H, KO525-A, KO252-H, mCherry-A, mCherry-H, PI-A, PI-H, and FSC-Width). These parameters were used as classifiers by CellScanner (see below). During the study period, the instrument was calibrated daily with CytoFLEX Daily QC Fluorospheres. A blank control containing only medium compared to SYBR positive communities was used to gate for total cell counts in the FL1-A channel, resulting in a threshold on FL1-A of ± 3,5*10^3^.

All the events were quantified using a volumetric method (events/μl). The same cell counts were used for calculating the absolute abundances of the three 16S rRNA gene sequencing replicates. We measured the technical replicates of two dilutions (100x and 1000x) of different species for two different timepoints, ± 20 minutes apart (Supplementary Table S3).

### Metabolite measurements

Frozen supernatant was thawed from −80°C (30 minutes, room temperature). Concentrations of trehalose, glucose, pyruvic acid, succinic acid, lactic acid, formic acid, acetic acid, propionic acid, iso-butyric acid, butyric acid, and iso-valeric acid in the supernatant were measured on an Agilent 1200 HPLC (Diegem, Belgium) system equipped with an Aminex HPX-87 H column (Bio-rad, Temse, Belgium). The column was kept at 40°C and eluted with 5 mM H_2_SO_4_ at a rate of 0.60 mL/min. Organic- and fatty acids were detected with UV (Agilent DAD, at 210 nm) and refractive index changes (Agilent RID, at 40°C). Trehalose and glucose were detected with RID only.^40^

### 16S rRNA gene sequencing and data processing

Extraction of DNA was carried out following an adapted protocol described by Falony *et al*. using the MagAttract PowerMicrobiome DNA/RNA Kit KF (Qiagen) according to the manufacturer’s instructions with the addition of a heating step (10 minutes at 90°C) after bead beating to increase DNA yield and removal of β-mercaptoethanol.^41^ The DNA was extracted and purified by the freedom evo TECAN extraction platform (Tecan, Switzerland) by means of several washing steps using ClearMag magnetic particles. Subsequently, a primer pair 515 F (5’ GTGYCAGCMGCCGCGGTAA 3’)/806 R (5’ GGACTACNVGGGTWTCTAAT 3’) was used to amplify the V4 region of the 16S rRNA gene by adding adapters and barcodes for sequencing. Sequencing was performed using the Illumina MiSeq platform to generate paired-end reads of 250 base pairs.

After demultiplexing with the bioinformatics tool sdm (simple demultiplexer) as part of the LotuS pipeline without allowing for mismatches, fastq sequences were preprocessed using DADA2 pipeline v1.14.1.^42,43^ The forward and reverse fastq files were submitted to ENA with the accession number PRJEB51873.

The taxonomy was assigned initially using the RDP classifier v2.13,^44^ but for taxa that were not correctly identified (e.g. only identified up to UC_g or UC_f, see Supplementary Table S4) the sequence variants were aligned using the BLAST Sequence Analysis Tool with refseq_rna to ensure accurate assignment of the species.^45^ Since our community is defined, we excluded species that were not community members assuming that they were contaminants introduced by the 16S rRNA gene sequencing pipeline, unless they were identified in more than one sample (Pseudomonas in the first experiment, see Supplementary Figure 3).

Taxon counts were corrected with 16S rRNA gene copy numbers of the exact strains retrieved from the rRNA operon copy number database rrnDB, and the National Center for Biotechnology Information (NCBI, Bethesda (MD): National Library of Medicine (US)) by looking at the whole-genome sequence summary (e.g. https://www.ncbi.nlm.nih.gov/nuccore/223987233). Sequencing data were converted to relative abundances and multiplied by the total cell count of the sample obtained by flow cytometry to obtain absolute abundance.^46^

Three technical replicates of 16S rRNA gene sequencing were performed in this study following the pipeline described above. The first technical replicate was prepared and sequenced separately from the other technical replicates. The second and third technical replicates were later processed together on the same plate during extraction, PCR and 16S rRNA sequencing runs. Due to the different placement of the six blank wells in the extraction- and PCR plates, the samples did not have the same neighboring samples as their technical replicates. The samples were handled by the same persons for all three technical replicates to minimize protocol bias.

### Event classification in mixed cultures with CellScanner

CellScanner is a standalone tool (manuscript submitted), which relies on supervised classification to train classifiers on flow cytometry data of monocultures to assign events in mixed cultures to species. The same principle is also used to automatically gate raw flow cytometry data. This gating strategy was used for all CellScanner data presented in this paper.

Machine gating was performed by training six random forest classifiers on flow cytometry data of monocultures and the blank vessel, respectively. These classifiers then recognize events as either cells or debris. Next, 10 random forest classifiers were trained and tested on 5000 random events for each gated monoculture dataset (4286 events for training and 714 events for testing). Next, each classifier predicts the species to which an event belongs in the community. When at least seven classifiers agree on a species, the event is classified as that species, else it is labeled as unknown. The monoculture of PC was already contaminated at the first measured timepoint, and since in addition PC was not consistently found in the 16S rRNA sequencing data, it was left out of the CellScanner analysis. Since we only had non-contaminated monocultures available at 67 h for RI and BH, we used monoculture data from 12 h for BT and CA and monoculture data from matching time points for RI and BH. In fact, by selecting monoculture datasets from different time points for the training, this grouping/combination resulted in the highest accuracy (assessed in silico) with fewer events assigned as unknown (Supplementary Figure 12).

### Statistical analyses

The PCoA plots were computed with R package vegan 2.5.7 using Bray Curtis dissimilarity and vegan’s envfit function to include metabolite data. R packages ggplot2 (3.3.6) and ggpubr (0.4.0) were used to create violin plots.

Heterogeneity in flow cytometry data was computed as the sum of the mean range for each channel across all events after gating.

## Supplementary Material

Supplemental Material

## Data sharing statement

The 16S rRNA gene sequencing data have been submitted to ENA with accession number PRJEB51873 and will be made available upon acceptance of this work. The processed data and R code for the figures is available at: http://msysbiology.com/supplements.html#variability CellScanner is available at: http://msysbiology.com/cellscanner.html.
