# Supplementary material for: Technical versus biological variability in a synthetic human gut community
Source: Gut Microbes. 2022 Dec 29;15(1):2155019. doi: 10.1080/19490976.2022.2155019 (PMC9809966; doi:10.1080/19490976.2022.2155019)

## Slide 1
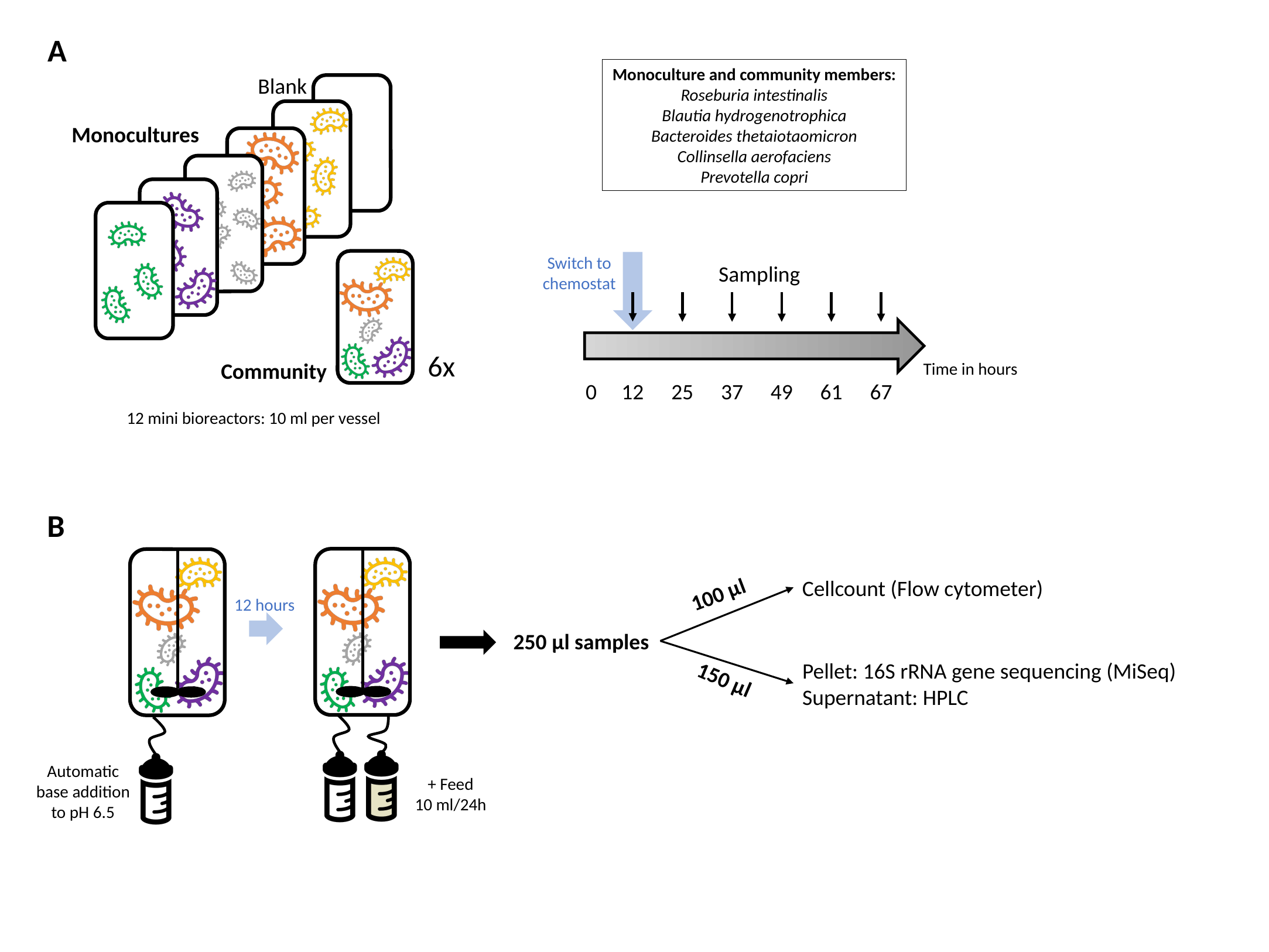

A
Monoculture and community members:
Roseburia intestinalis
Blautia hydrogenotrophica
Bacteroides thetaiotaomicron
Collinsella aerofaciens
Prevotella copri
Blank
Monocultures
Community
12 mini bioreactors: 10 ml per vessel
6x
Switch to chemostat
Sampling
Time in hours
0
12
25
37
49
61
67
B
Cellcount (Flow cytometer)
100 μl
250 μl samples
Pellet: 16S rRNA gene sequencing (MiSeq)
Supernatant: HPLC
150 μl
12 hours
Automatic base addition
to pH 6.5
+ Feed
10 ml/24h

## Slide 2
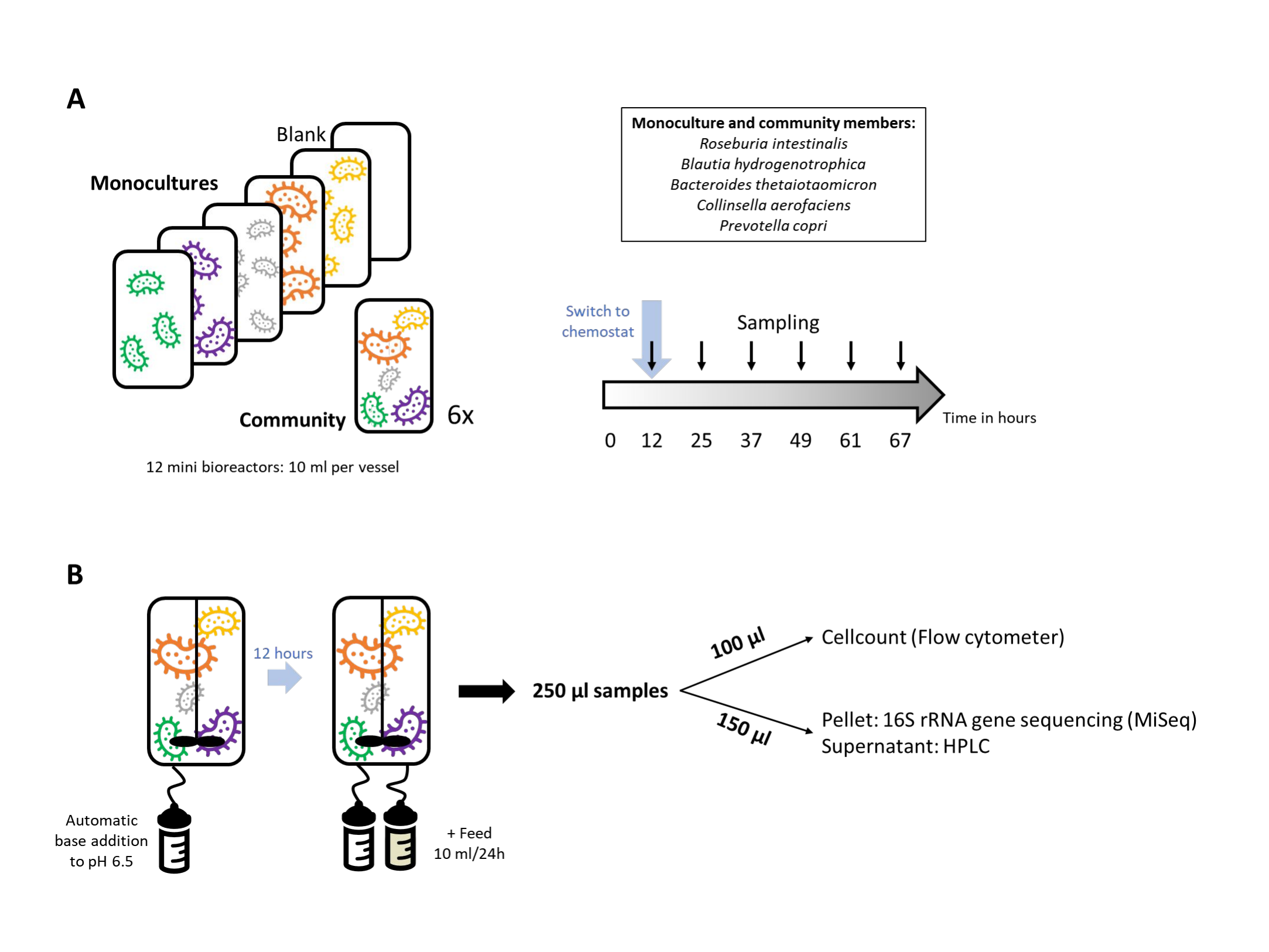

## Slide 3
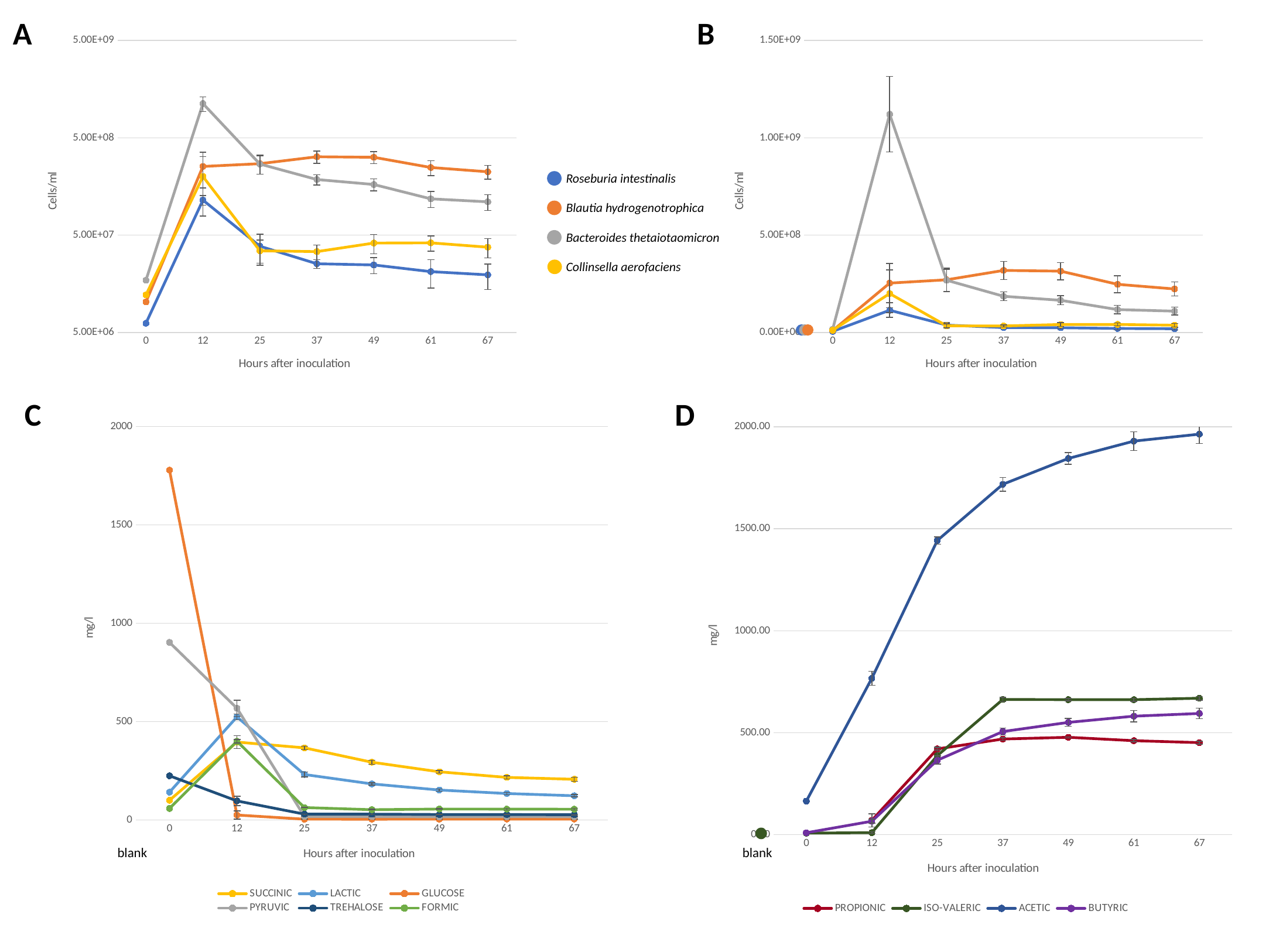

A
B
### Chart
| Category | Roseburia intestinalis | Blautia hydrogenotrophica | Bacteroides thetaiotaomicron | Collinsella aerofaciens |
|---|---|---|---|---|
| 0 | 6236000.0 | 10300200.0 | 17227600.0 | 12156000.0 |
| 12 | 114559398.16314553 | 253874060.96642867 | 1120815669.9402304 | 199977655.46192193 |
| 25 | 38475186.71679466 | 270871701.07439715 | 268506867.1648348 | 34542248.832830064 |
| 37 | 25433355.57140872 | 318991696.2871862 | 186278429.82118884 | 33882193.9054469 |
| 49 | 24724219.977240413 | 315234252.8751169 | 165706675.86979267 | 41473642.11098812 |
| 61 | 21114165.762939345 | 247673492.21652636 | 117972793.59329319 | 41670048.427240886 |
| 67 | 19572967.925248686 | 223842284.1511723 | 109968576.59348698 | 37625774.77207978 |
### Chart
| Category | Roseburia intestinalis | Blautia hydrogenotrophica | Bacteroides thetaiotaomicron | Collinsella aerofaciens |
|---|---|---|---|---|
| 0 | 6236000.0 | 10300200.0 | 17227600.0 | 12156000.0 |
| 12 | 114559398.16314553 | 253874060.96642867 | 1120815669.9402304 | 199977655.46192193 |
| 25 | 38475186.71679466 | 270871701.07439715 | 268506867.1648348 | 34542248.832830064 |
| 37 | 25433355.57140872 | 318991696.2871862 | 186278429.82118884 | 33882193.9054469 |
| 49 | 24724219.977240413 | 315234252.8751169 | 165706675.86979267 | 41473642.11098812 |
| 61 | 21114165.762939345 | 247673492.21652636 | 117972793.59329319 | 41670048.427240886 |
| 67 | 19572967.925248686 | 223842284.1511723 | 109968576.59348698 | 37625774.77207978 |Roseburia intestinalis
Blautia hydrogenotrophica
Bacteroides thetaiotaomicron
Collinsella aerofaciens
C
D
### Chart
| Category | SUCCINIC | LACTIC | GLUCOSE | PYRUVIC | TREHALOSE | FORMIC |
|---|---|---|---|---|---|---|
| 0 | 99.77 | 141.0 | 1778.48 | 902.69 | 224.4 | 58.22 |
| 12 | 395.6916666666666 | 523.7016666666667 | 24.915000000000003 | 567.7533333333333 | 96.61666666666667 | 399.94166666666666 |
| 25 | 366.34666666666664 | 230.92666666666665 | 3.73 | 17.634999999999998 | 29.473333333333333 | 62.794999999999995 |
| 37 | 293.26 | 183.08333333333337 | 3.6200000000000006 | 16.724999999999998 | 29.325000000000003 | 51.893333333333324 |
| 49 | 244.57500000000005 | 152.045 | 3.8925 | 16.09 | 27.429999999999996 | 55.294999999999995 |
| 61 | 216.09833333333333 | 134.18833333333333 | 4.265000000000001 | 16.351666666666667 | 27.146666666666672 | 54.88833333333333 |
| 67 | 206.705 | 122.83999999999999 | 4.3740000000000006 | 16.3 | 26.46666666666667 | 54.39833333333333 |blank
### Chart
| Category | PROPIONIC | ISO-VALERIC | ACETIC | BUTYRIC |
|---|---|---|---|---|
| 0 | None | 7.87 | 164.91 | 9.59 |
| 12 | 70.45333333333333 | 10.028333333333332 | 766.9616666666667 | 66.02333333333333 |
| 25 | 420.67333333333335 | 387.83666666666664 | 1442.945 | 365.2 |
| 37 | 469.21166666666664 | 663.12 | 1718.3216666666667 | 505.8883333333333 |
| 49 | 477.6666666666667 | 662.2366666666667 | 1845.1066666666666 | 550.65 |
| 61 | 461.12166666666667 | 662.0516666666666 | 1930.1283333333333 | 581.23 |
| 67 | 451.5416666666667 | 669.1566666666666 | 1964.2066666666667 | 594.4466666666666 |
blank

## Slide 4
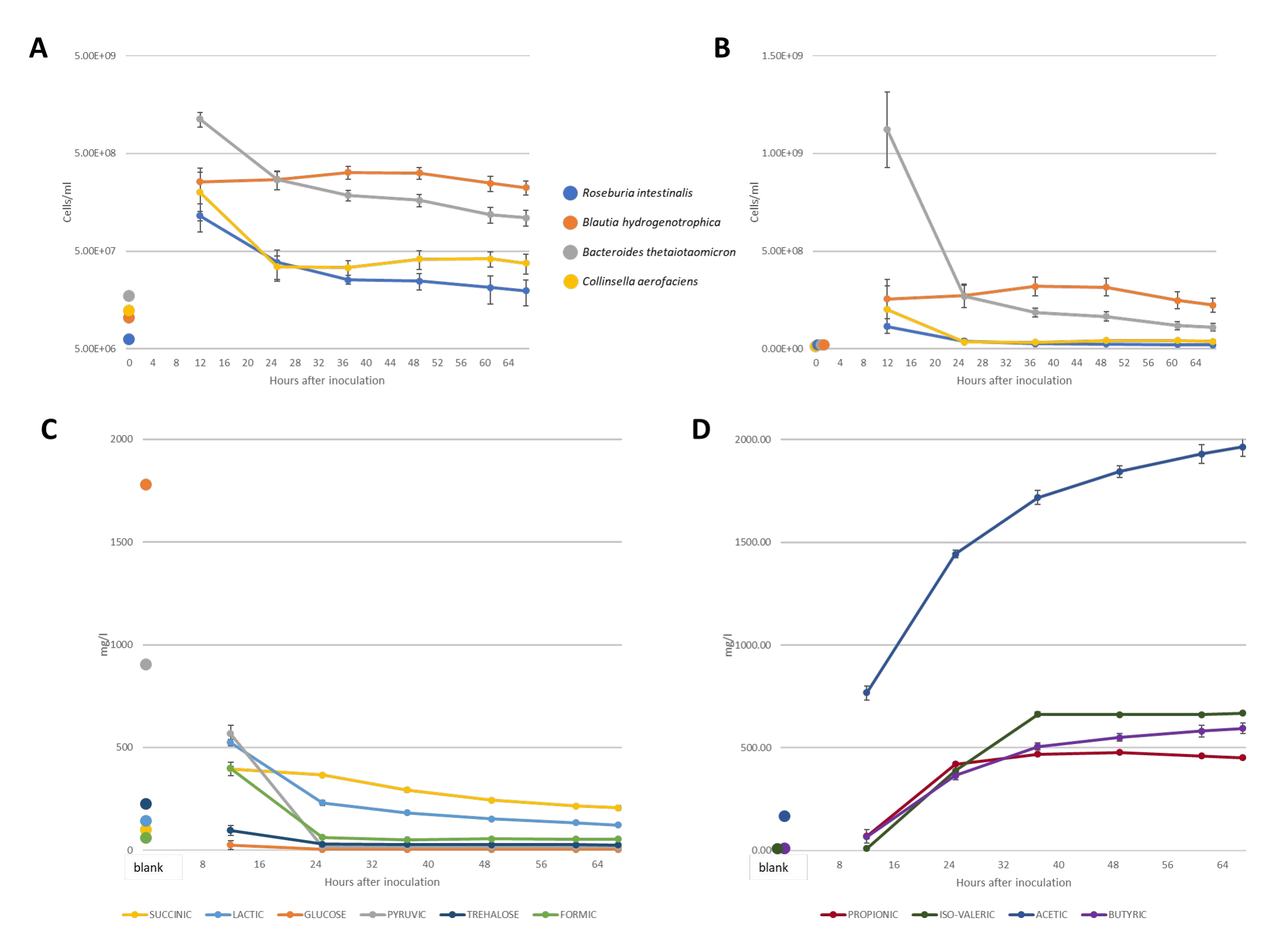

## Slide 5
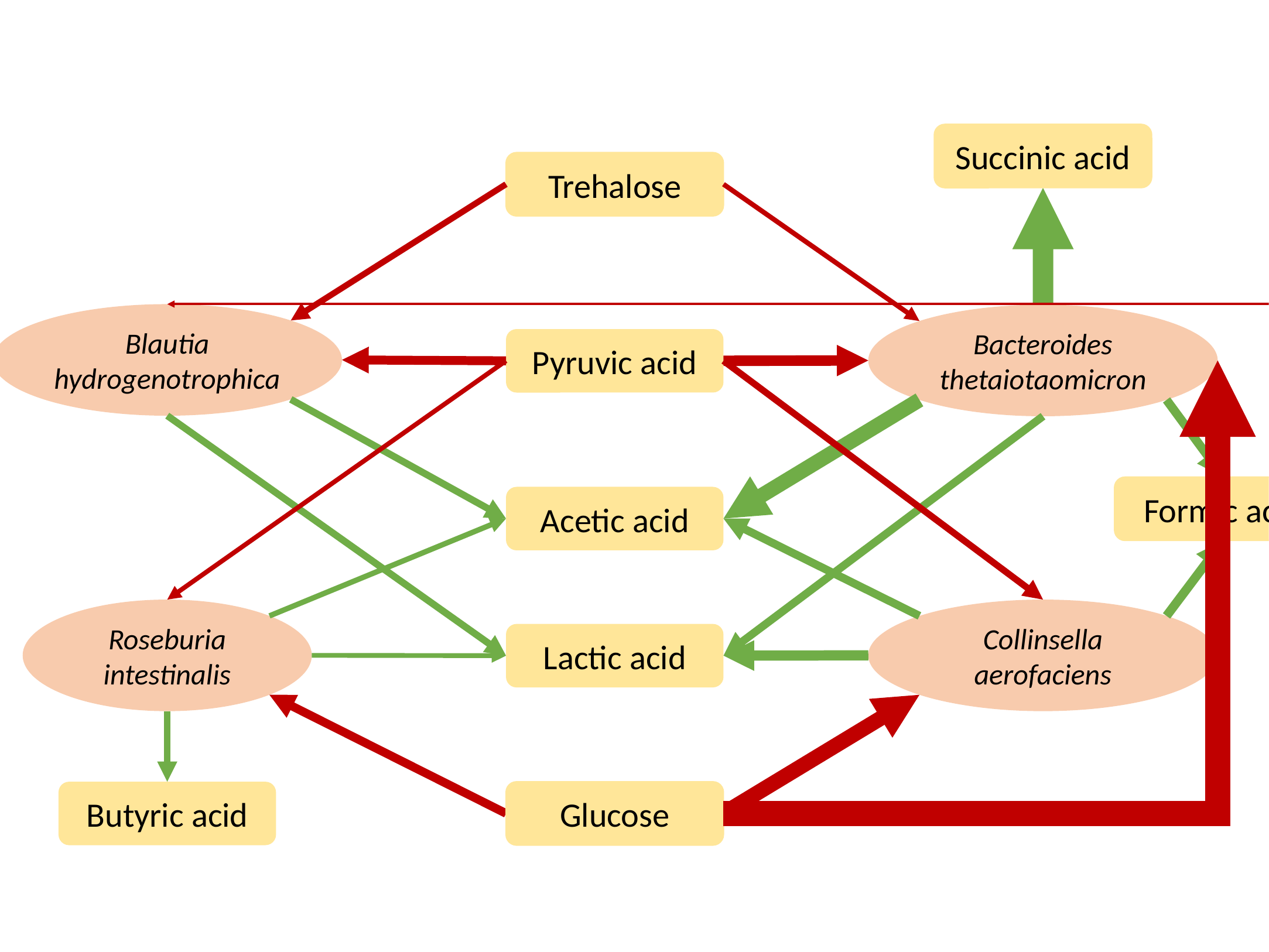

Succinic acid
Trehalose
Blautia hydrogenotrophica
Bacteroides thetaiotaomicron
Pyruvic acid
Formic acid
Acetic acid
Roseburia intestinalis
Collinsella aerofaciens
Lactic acid
Butyric acid
Glucose

## Slide 6
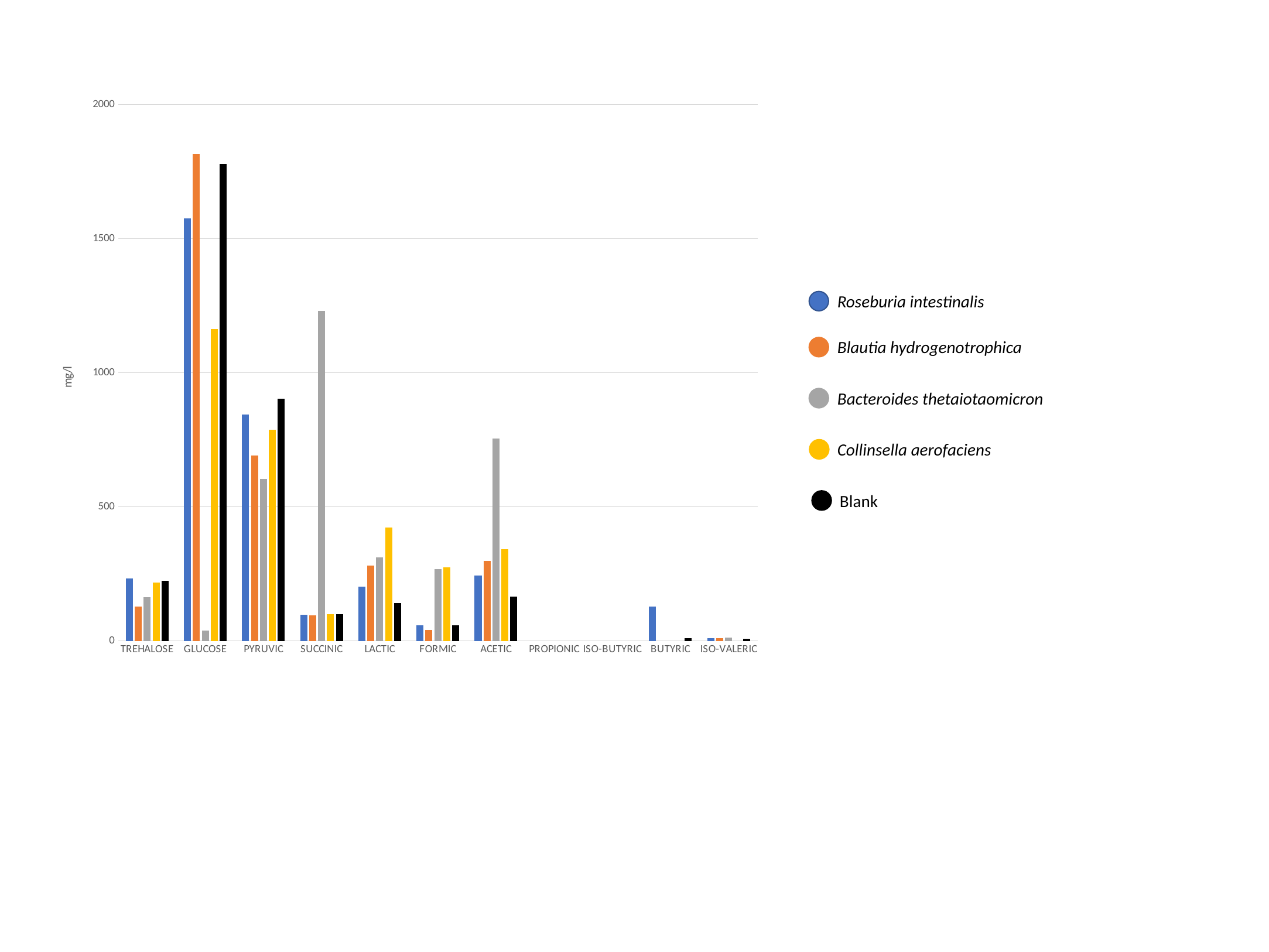

### Chart
| Category | | | | | |
|---|---|---|---|---|---|
| TREHALOSE | 233.75 | 126.97 | 162.29 | 216.59 | 224.4 |
| GLUCOSE | 1575.15 | 1816.15 | 38.19 | 1162.65 | 1778.48 |
| PYRUVIC | 845.04 | 691.99 | 604.86 | 787.6 | 902.69 |
| SUCCINIC | 96.88 | 95.8 | 1231.54 | 99.35 | 99.77 |
| LACTIC | 202.96 | 280.33 | 310.89 | 422.67 | 141.0 |
| FORMIC | 58.14 | 41.23 | 266.63 | 273.52 | 58.22 |
| ACETIC | 243.26 | 297.89 | 754.82 | 343.06 | 164.91 |
| PROPIONIC | None | None | None | None | None |
| ISO-BUTYRIC | None | None | None | None | None |
| BUTYRIC | 128.49 | None | None | None | 9.59 |
| ISO-VALERIC | 9.04 | 10.58 | 13.21 | None | 7.87 |Roseburia intestinalis
Blautia hydrogenotrophica
Bacteroides thetaiotaomicron
Collinsella aerofaciens
Blank

## Slide 7
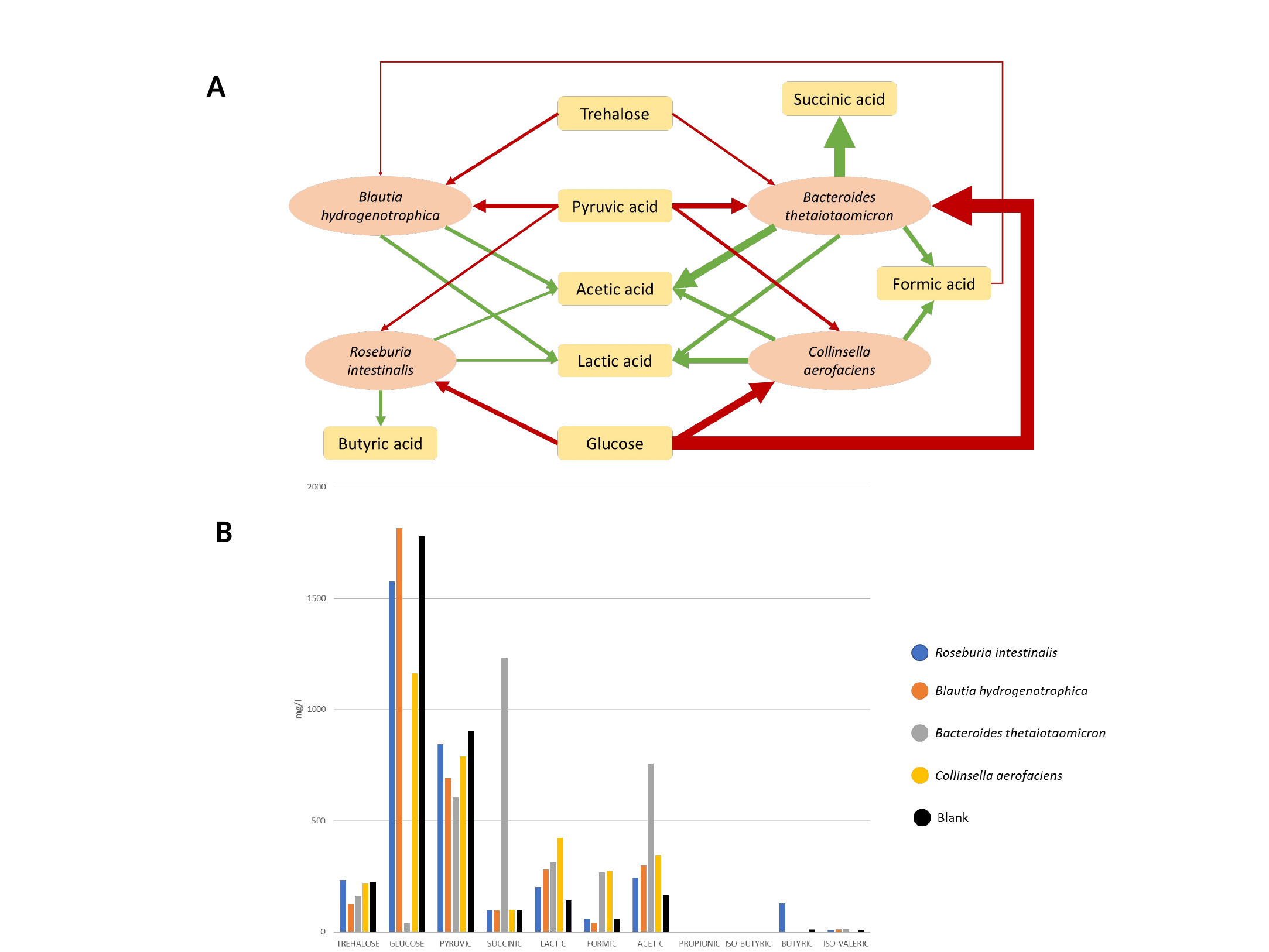

A
B

## Slide 8
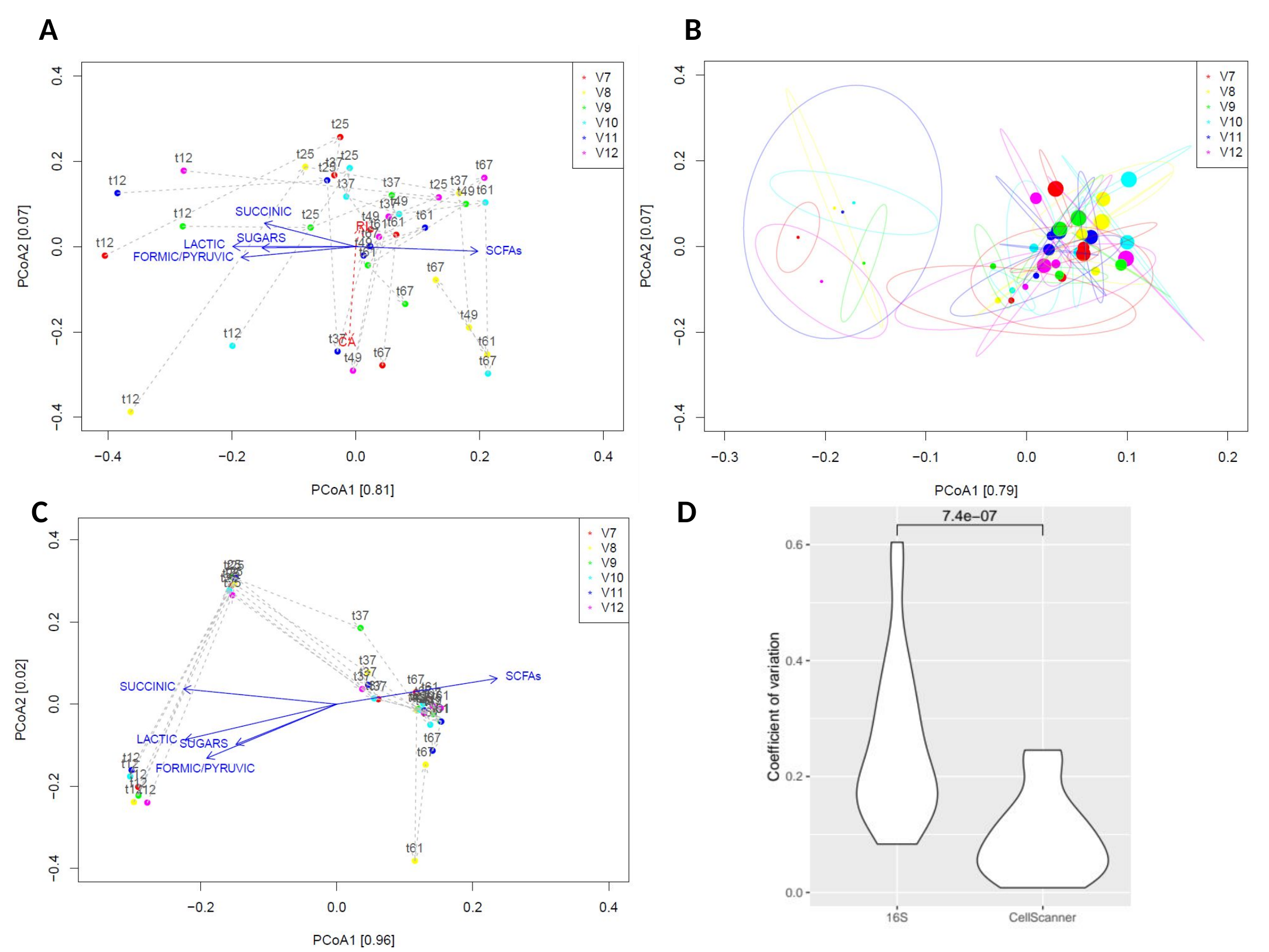

A
B
C
D

## Slide 9
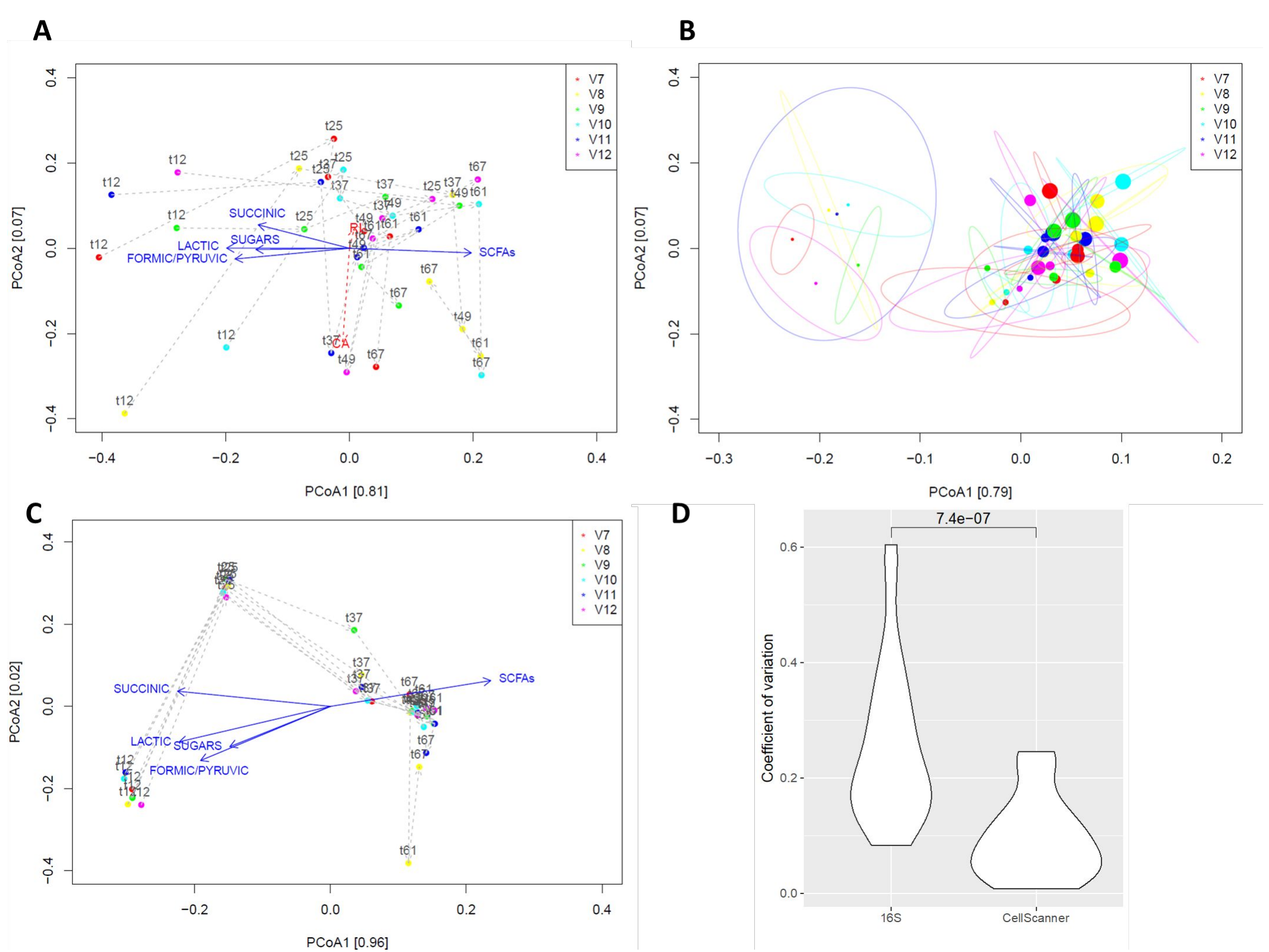

## Slide 10
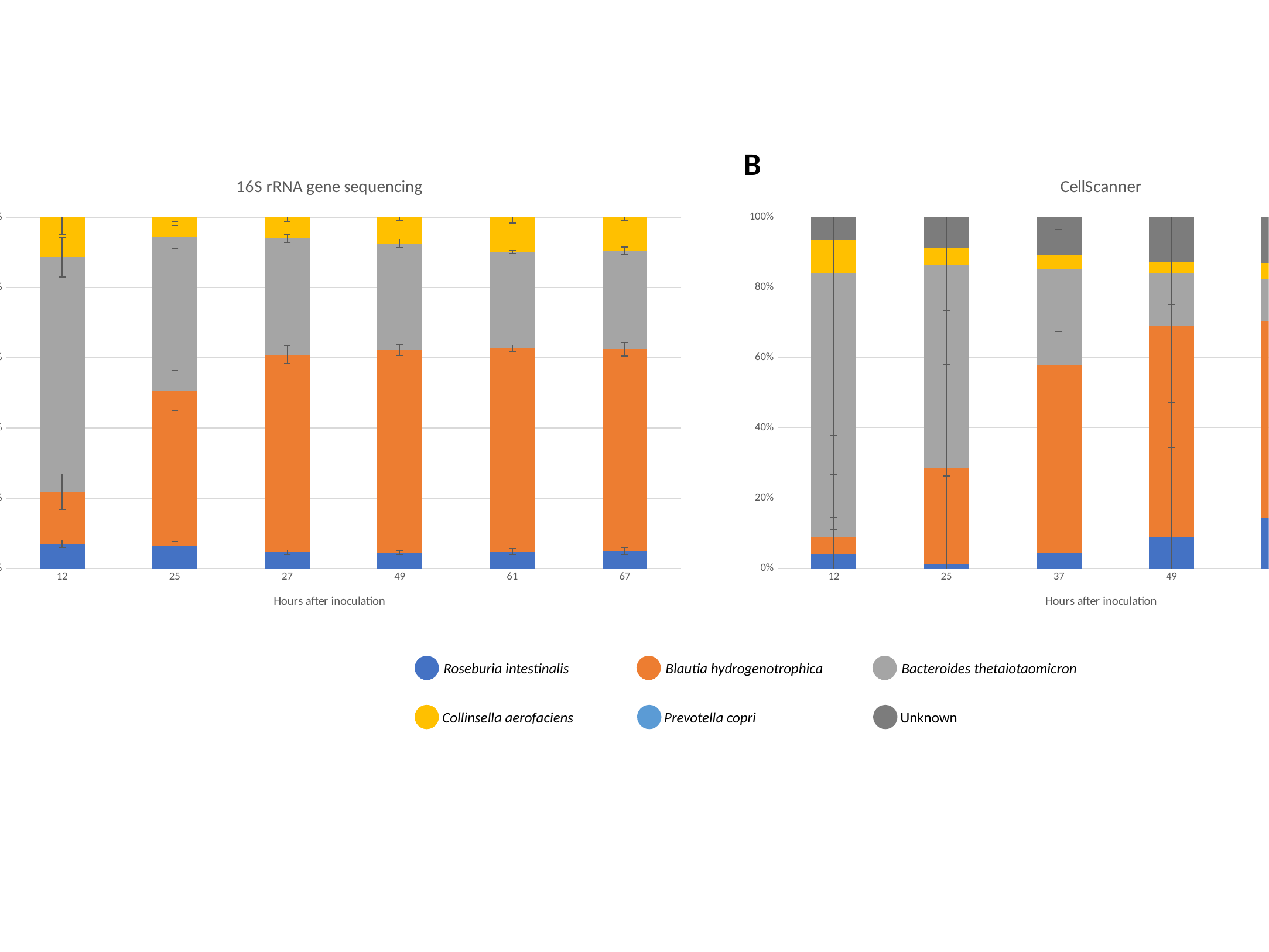

A
B
### Chart: CellScanner
| Category | Roseburia intestinalis | Blautia hydrogenotrophica | Bacteroides thetaiotaomicron | Collinsella aerofaciens | unknown |
|---|---|---|---|---|---|
| 12 | 3.9759744862587945 | 4.943003480593883 | 75.22360440795669 | 9.307510530871918 | 6.549907094318719 |
| 25 | 1.170644665958712 | 27.201188637250283 | 58.086821649859694 | 4.789010849343843 | 8.75233419758746 |
| 37 | 4.295033321509571 | 53.58534731101821 | 27.221207533933384 | 3.922567087793926 | 10.975844745744906 |
| 49 | 8.962060064799047 | 59.92027315101831 | 15.006338042560152 | 3.421461495835723 | 12.689867245786772 |
| 61 | 14.251840278828107 | 56.24902985136236 | 11.70965084713289 | 4.598296411348048 | 13.191182611328601 |
| 67 | 16.55700319058251 | 54.76807826655997 | 12.82140955215373 | 4.619525673609643 | 11.233983317094149 |
### Chart: 16S rRNA gene sequencing
| Category | Roseburia intestinalis | Blautia hydrogenotrophica | Bacteroides thetaiotaomicron | Collinsella aerofaciens | Prevotella copri |
|---|---|---|---|---|---|
| 12 | 0.06958063648542633 | 0.1485138050869199 | 0.6681815482254173 | 0.1136157071180149 | 8.73960681755259e-05 |
| 25 | 0.06220549978877227 | 0.444006036183273 | 0.43707443368583004 | 0.056675896051947276 | 2.9732107264703216e-05 |
| 27 | 0.04563523190677831 | 0.563091618009622 | 0.33000871425756784 | 0.06125676886608939 | 3.2452626737243166e-06 |
| 49 | 0.045310863560821736 | 0.576385748070118 | 0.30324582240167036 | 0.07504767954813348 | 4.09279177512565e-06 |
| 61 | 0.04830444614257984 | 0.5778625469147018 | 0.2746756539172928 | 0.09915735302542568 | 0.0 |
| 67 | 0.049668696393102056 | 0.5741453534094113 | 0.28075579402330436 | 0.09542675686765485 | 0.0 |Roseburia intestinalis
Blautia hydrogenotrophica
Bacteroides thetaiotaomicron
Collinsella aerofaciens
Prevotella copri
Unknown

## Slide 11
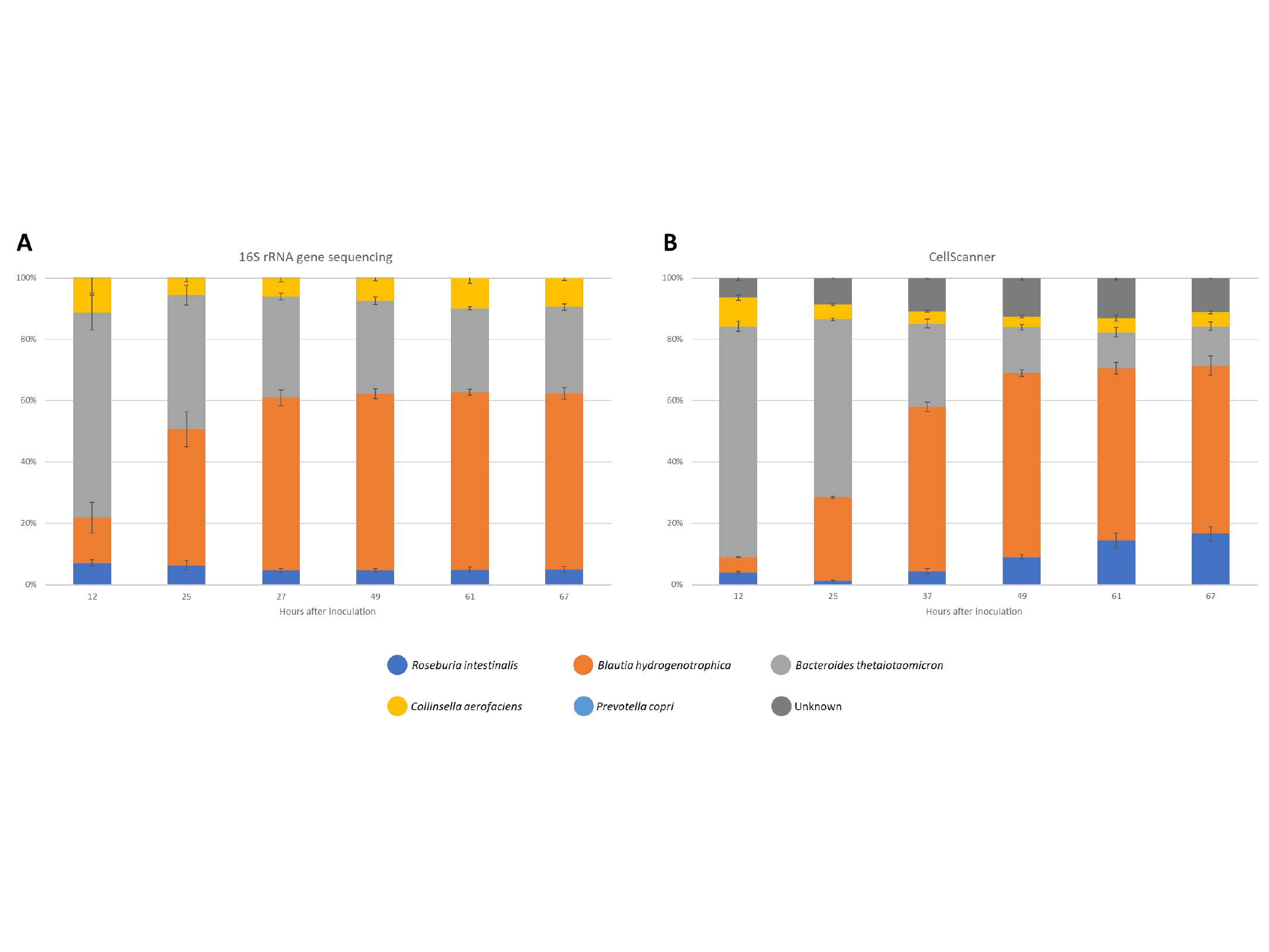

## Slide 12
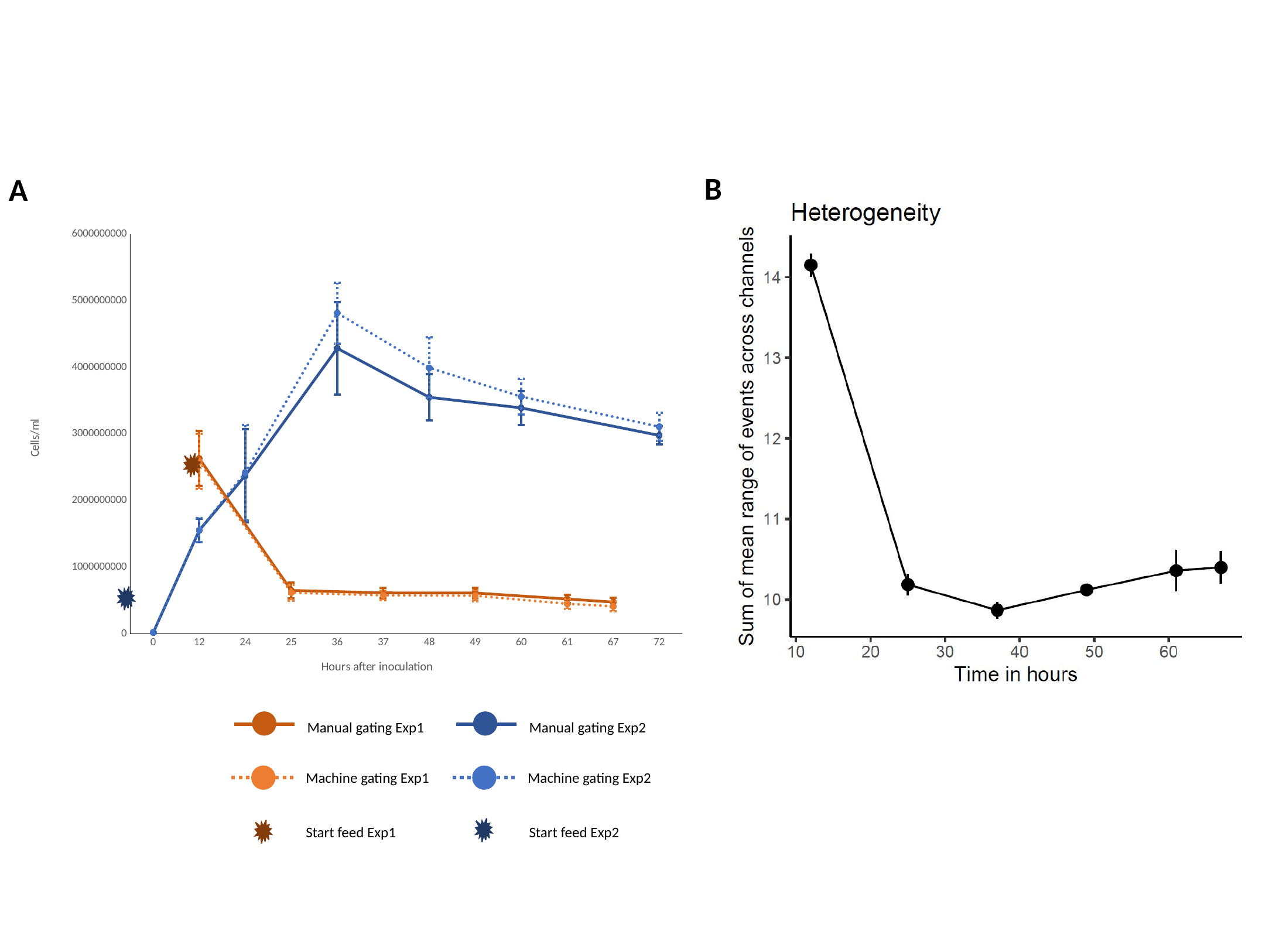

B
A
### Chart
| Category | Manual gating Exp2 | CellScanner gating Exp2 | Manual gating Exp1 | CellScanner gating Exp1 |
|---|---|---|---|---|
| 0 | 17293666.666666668 | 20807076.290078644 | None | None |
| 12 | 1551863666.6666667 | 1554743211.5733335 | 2632583166.6666665 | 2586218014.856667 |
| 24 | 2373655000.0 | 2419107642.766667 | None | None |
| 25 | None | None | 650522666.6666666 | 616818480.505524 |
| 36 | 4286575666.6666665 | 4816725594.533334 | None | None |
| 37 | None | None | 614667833.3333334 | 576996950.7692441 |
| 48 | 3550597666.6666665 | 3993465672.5733337 | None | None |
| 49 | None | None | 612119500.0 | 571589558.8220904 |
| 60 | 3392159666.6666665 | 3561459014.2333336 | None | None |
| 61 | None | None | 521527000.0 | 449884580.512361 |
| 67 | None | None | 475492333.3333333 | 412247732.98381215 |
| 72 | 2978742333.3333335 | 3108724610.4733334 | None | None |
Manual gating Exp1
Manual gating Exp2
Machine gating Exp1
Machine gating Exp2
Start feed Exp1
Start feed Exp2

## Slide 13
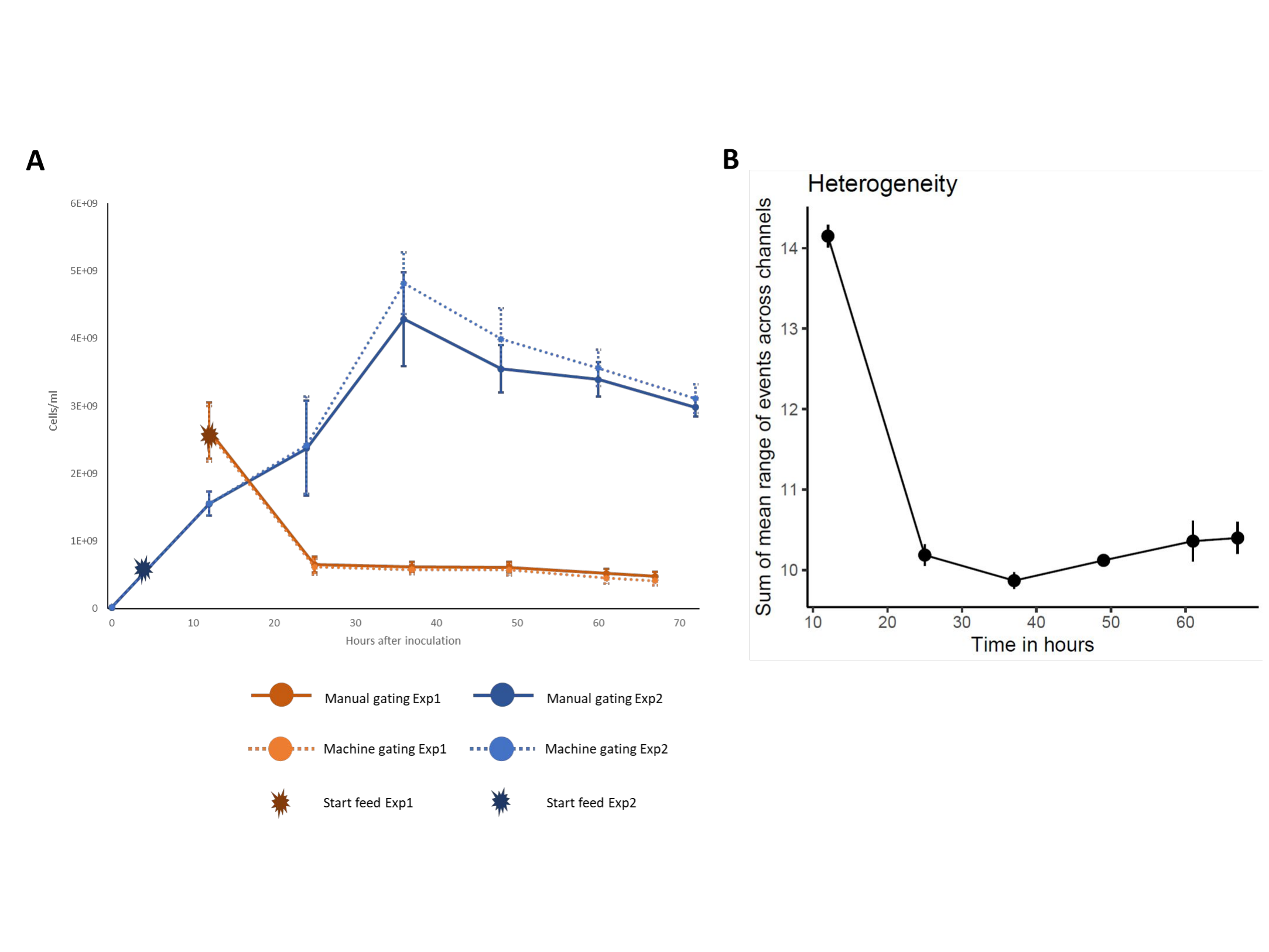

Supplement: Supplemental Material [file KGMI_A_2155019_SM3088.zip › Revised_Figures_Complete.pptx]
