# Supplementary material for: Technical versus biological variability in a synthetic human gut community
Source: Gut Microbes. 2022 Dec 29;15(1):2155019. doi: 10.1080/19490976.2022.2155019 (PMC9809966; doi:10.1080/19490976.2022.2155019)
Supplement: Supplemental Material [file KGMI_A_2155019_SM3088.zip › Revised_SupplementaryFigures_Complete.pptx]

## Slide 1
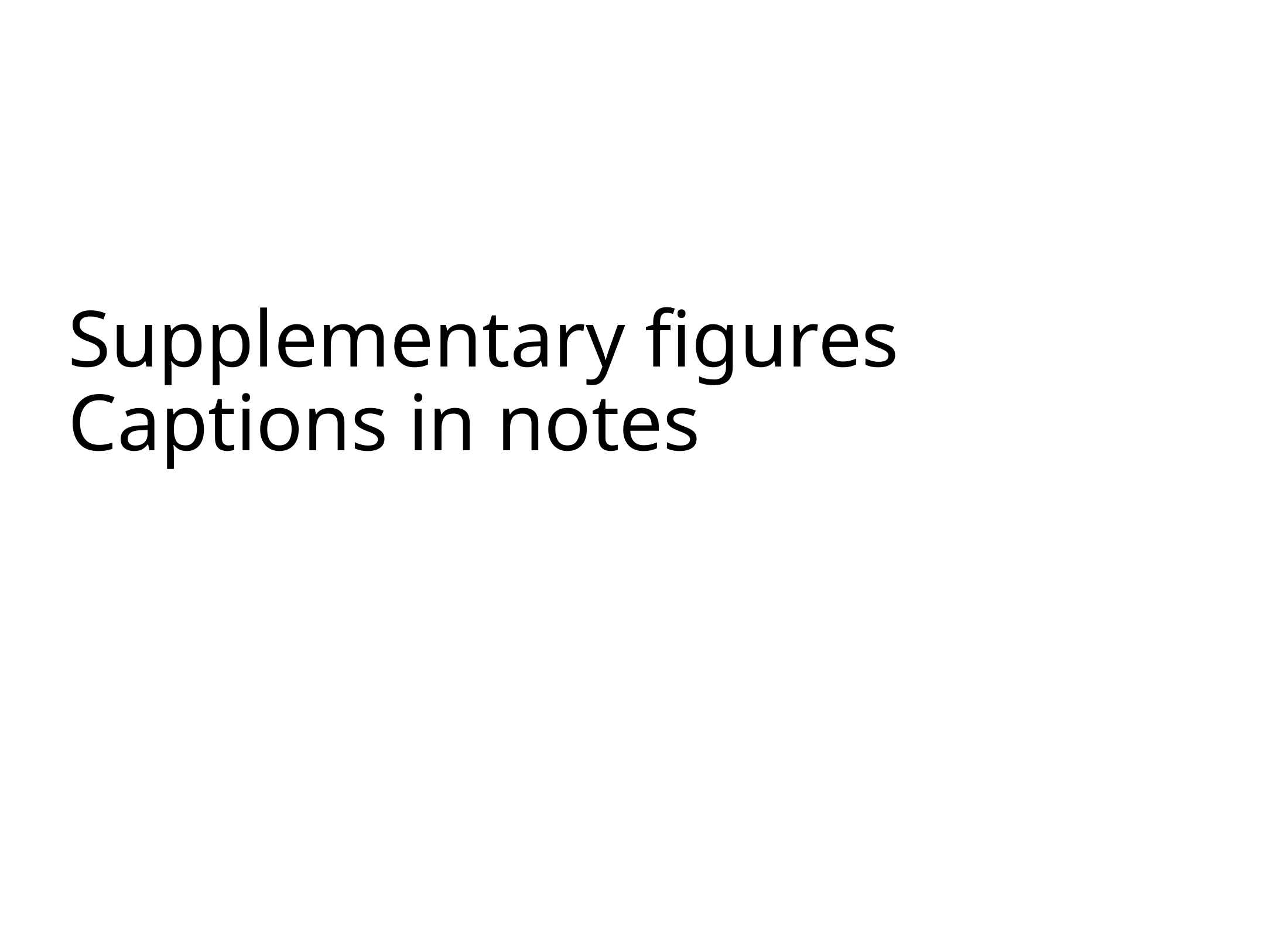

# Supplementary figures Captions in notes

## Slide 2
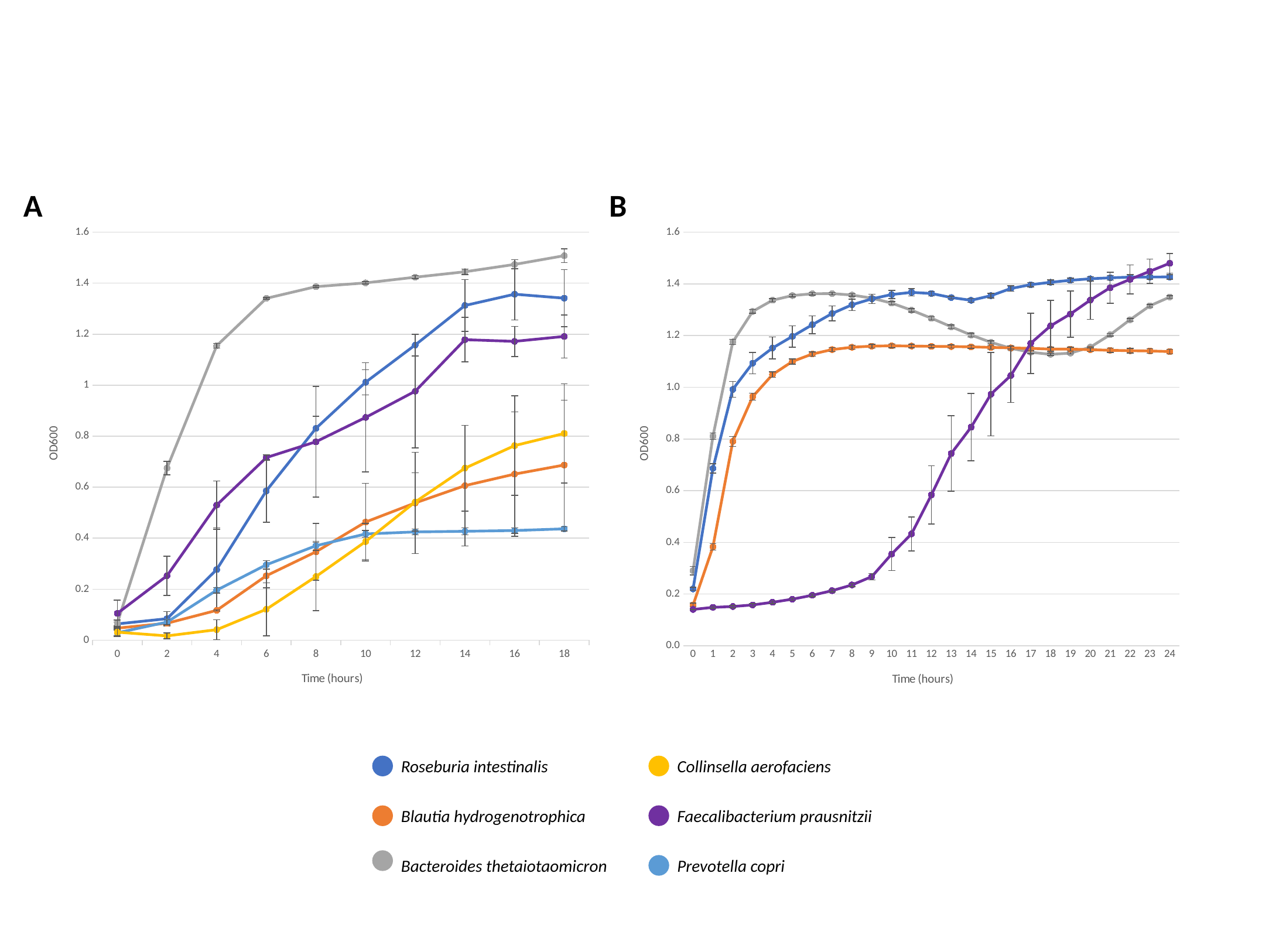

A
B
### Chart
| Category | RI | BH | BT | FP | PC | CA |
|---|---|---|---|---|---|---|
| 0 | 0.06400000000000006 | 0.04733333333333334 | 0.06699999999999995 | 0.10533333333333333 | 0.02899999999999997 | 0.03151075268817157 |
| 2 | 0.08433333333333332 | 0.0663333333333333 | 0.6746666666666667 | 0.25266666666666665 | 0.071 | 0.016844086021504907 |
| 4 | 0.27699999999999997 | 0.11699999999999999 | 1.1546666666666667 | 0.5296666666666666 | 0.19599999999999995 | 0.04117741935483824 |
| 6 | 0.5856666666666667 | 0.2526666666666666 | 1.3406666666666667 | 0.7163333333333334 | 0.29566666666666663 | 0.12117741935483822 |
| 8 | 0.831 | 0.3466666666666666 | 1.3866666666666665 | 0.7783333333333333 | 0.37033333333333335 | 0.2495107526881716 |
| 10 | 1.0116666666666667 | 0.4633333333333333 | 1.4013333333333335 | 0.8736666666666667 | 0.4166666666666667 | 0.38684408602150494 |
| 12 | 1.158 | 0.5383333333333333 | 1.4236666666666664 | 0.9766666666666667 | 0.4246666666666667 | 0.5421774193548382 |
| 14 | 1.3126666666666669 | 0.6059999999999999 | 1.4449999999999998 | 1.1786666666666665 | 0.427 | 0.6745107526881715 |
| 16 | 1.357 | 0.6513333333333334 | 1.4736666666666667 | 1.172 | 0.42999999999999994 | 0.7631774193548382 |
| 18 | 1.341333333333333 | 0.6869999999999999 | 1.508 | 1.1913333333333334 | 0.4366666666666666 | 0.8108440860215049 |
### Chart
| Category | | | | |
|---|---|---|---|---|
| 0 | 0.29066666666666663 | 0.22 | 0.15766666666666665 | 0.14066666666666663 |
| 1 | 0.8106666666666666 | 0.6863333333333335 | 0.3826666666666666 | 0.14866666666666664 |
| 2 | 1.1753333333333333 | 0.992 | 0.7906666666666666 | 0.15200000000000005 |
| 3 | 1.2936666666666667 | 1.0936666666666668 | 0.9636666666666667 | 0.15799999999999995 |
| 4 | 1.337 | 1.1520000000000001 | 1.0493333333333332 | 0.16833333333333336 |
| 5 | 1.3549999999999998 | 1.197 | 1.0999999999999999 | 0.18033333333333337 |
| 6 | 1.362 | 1.242 | 1.1286666666666667 | 0.1953333333333334 |
| 7 | 1.3626666666666667 | 1.2856666666666667 | 1.146 | 0.2133333333333333 |
| 8 | 1.357 | 1.3193333333333335 | 1.155 | 0.2353333333333333 |
| 9 | 1.3449999999999998 | 1.3423333333333334 | 1.1593333333333333 | 0.2676666666666666 |
| 10 | 1.3256666666666665 | 1.359 | 1.1606666666666665 | 0.3550000000000001 |
| 11 | 1.2983333333333331 | 1.367333333333333 | 1.1596666666666666 | 0.43300000000000005 |
| 12 | 1.2673333333333332 | 1.3629999999999998 | 1.1586666666666667 | 0.5836666666666668 |
| 13 | 1.2343333333333335 | 1.3473333333333333 | 1.1576666666666666 | 0.744 |
| 14 | 1.2023333333333335 | 1.3366666666666667 | 1.1566666666666665 | 0.846 |
| 15 | 1.1739999999999997 | 1.3546666666666667 | 1.1543333333333334 | 0.9736666666666668 |
| 16 | 1.151 | 1.3823333333333332 | 1.1526666666666667 | 1.0456666666666665 |
| 17 | 1.1356666666666666 | 1.397 | 1.1506666666666667 | 1.1703333333333334 |
| 18 | 1.1276666666666666 | 1.4063333333333332 | 1.148 | 1.2376666666666667 |
| 19 | 1.1323333333333332 | 1.4140000000000001 | 1.1480000000000001 | 1.2836666666666667 |
| 20 | 1.1553333333333335 | 1.42 | 1.1456666666666668 | 1.3373333333333335 |
| 21 | 1.2033333333333336 | 1.4236666666666666 | 1.1433333333333333 | 1.3853333333333333 |
| 22 | 1.2613333333333334 | 1.4263333333333335 | 1.1416666666666666 | 1.4176666666666664 |
| 23 | 1.3153333333333332 | 1.4266666666666667 | 1.1406666666666667 | 1.4490000000000003 |
| 24 | 1.349333333333333 | 1.4269999999999998 | 1.1383333333333334 | 1.4799999999999998 |Roseburia intestinalis
Collinsella aerofaciens
Blautia hydrogenotrophica
Faecalibacterium prausnitzii
Bacteroides thetaiotaomicron
Prevotella copri

## Slide 3
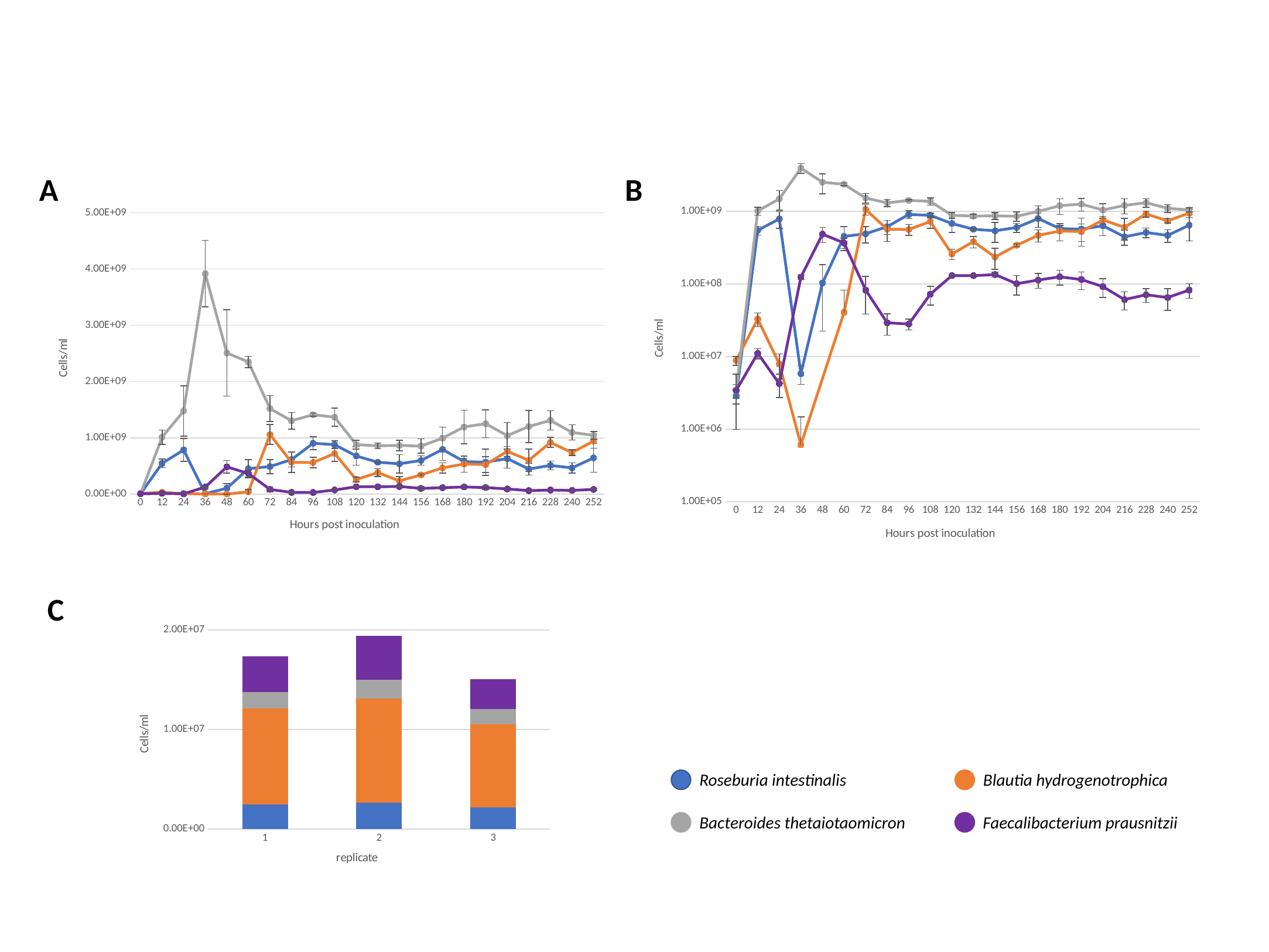

### Chart
| Category | Roseburia intestinalis | Blautia hydrogenotrophica | Bacteroides thetaiotaomicron | Faecalibacterium prausnitzii |
|---|---|---|---|---|
| 0 | 2879880.165166953 | 8777851.324396247 | 3363239.4114018134 | 3411590.9706170536 |
| 12 | 546322115.1228331 | 32822338.9785559 | 1010421315.7664427 | 11056563.465501746 |
| 24 | 783673756.6670628 | 7869248.6601284295 | 1477969051.741108 | 4220942.931700753 |
| 36 | 5799880.805523463 | 609857.0621194033 | 3918653669.289203 | 124116926.17648633 |
| 48 | 102752291.7368852 | 0.0 | 2509107277.323293 | 483538430.939821 |
| 60 | 450651028.48154265 | 40455203.33283939 | 2348907622.78297 | 365657812.06931597 |
| 72 | 489472544.6034846 | 1058536547.331401 | 1519466340.58481 | 82347900.81363817 |
| 84 | 612615525.078983 | 564270852.2692839 | 1302349523.1252635 | 29148432.85980257 |
| 96 | 903101699.4152414 | 560886607.4556997 | 1411110632.02867 | 27997727.767057136 |
| 108 | 877775826.3604673 | 720296806.6936306 | 1366594798.5164566 | 72107568.42944576 |
| 120 | 677323877.9408946 | 258293135.21066102 | 878762912.1830846 | 129856407.99869366 |
| 132 | 565219765.458527 | 381073541.8302593 | 857883812.1739584 | 129698213.87058835 |
| 144 | 537945191.505704 | 234104953.44258466 | 864014862.498891 | 134284659.21948698 |
| 156 | 597064220.97052 | 340037728.657609 | 852157349.3369986 | 100496367.7015387 |
| 168 | 793057012.5584415 | 465802096.87447166 | 993831298.2357883 | 112940592.33129935 |
| 180 | 577169888.8749672 | 533392788.655045 | 1193087717.2684987 | 124970605.20149066 |
| 192 | 565545763.7505223 | 524292161.51933736 | 1251767137.1203578 | 114630937.60978298 |
| 204 | 630805456.3888007 | 758174903.664403 | 1038594246.0191984 | 91576060.59426226 |
| 216 | 444152122.9068592 | 600747282.1684353 | 1201631683.164037 | 60725724.165444374 |
| 228 | 509631342.8176859 | 917327991.4027348 | 1309462530.4462233 | 70813325.34434913 |
| 240 | 464604682.0956588 | 737127341.1303946 | 1096169768.9155104 | 64850674.17558084 |
| 252 | 643094778.8256484 | 942137358.3619174 | 1043985470.2896305 | 82084280.4383105 |A
B
### Chart
| Category | Roseburia intestinalis | Blautia hydrogenotrophica | Bacteroides thetaiotaomicron | Faecalibacterium prausnitzii |
|---|---|---|---|---|
| 0 | 2879880.165166953 | 8777851.324396247 | 3363239.4114018134 | 3411590.9706170536 |
| 12 | 546322115.1228331 | 32822338.9785559 | 1010421315.7664427 | 11056563.465501746 |
| 24 | 783673756.6670628 | 7869248.6601284295 | 1477969051.741108 | 4220942.931700753 |
| 36 | 5799880.805523463 | 609857.0621194033 | 3918653669.289203 | 124116926.17648633 |
| 48 | 102752291.7368852 | 0.0 | 2509107277.323293 | 483538430.939821 |
| 60 | 450651028.48154265 | 40455203.33283939 | 2348907622.78297 | 365657812.06931597 |
| 72 | 489472544.6034846 | 1058536547.331401 | 1519466340.58481 | 82347900.81363817 |
| 84 | 612615525.078983 | 564270852.2692839 | 1302349523.1252635 | 29148432.85980257 |
| 96 | 903101699.4152414 | 560886607.4556997 | 1411110632.02867 | 27997727.767057136 |
| 108 | 877775826.3604673 | 720296806.6936306 | 1366594798.5164566 | 72107568.42944576 |
| 120 | 677323877.9408946 | 258293135.21066102 | 878762912.1830846 | 129856407.99869366 |
| 132 | 565219765.458527 | 381073541.8302593 | 857883812.1739584 | 129698213.87058835 |
| 144 | 537945191.505704 | 234104953.44258466 | 864014862.498891 | 134284659.21948698 |
| 156 | 597064220.97052 | 340037728.657609 | 852157349.3369986 | 100496367.7015387 |
| 168 | 793057012.5584415 | 465802096.87447166 | 993831298.2357883 | 112940592.33129935 |
| 180 | 577169888.8749672 | 533392788.655045 | 1193087717.2684987 | 124970605.20149066 |
| 192 | 565545763.7505223 | 524292161.51933736 | 1251767137.1203578 | 114630937.60978298 |
| 204 | 630805456.3888007 | 758174903.664403 | 1038594246.0191984 | 91576060.59426226 |
| 216 | 444152122.9068592 | 600747282.1684353 | 1201631683.164037 | 60725724.165444374 |
| 228 | 509631342.8176859 | 917327991.4027348 | 1309462530.4462233 | 70813325.34434913 |
| 240 | 464604682.0956588 | 737127341.1303946 | 1096169768.9155104 | 64850674.17558084 |
| 252 | 643094778.8256484 | 942137358.3619174 | 1043985470.2896305 | 82084280.4383105 |C
### Chart
| Category | Roseburia intestinalis | Blautia hydrogenotrophica | Bacteroides thetaiotaomicron | Faecalibacterium prausnitzii |
|---|---|---|---|---|Roseburia intestinalis
Blautia hydrogenotrophica
Faecalibacterium prausnitzii
Bacteroides thetaiotaomicron

## Slide 4
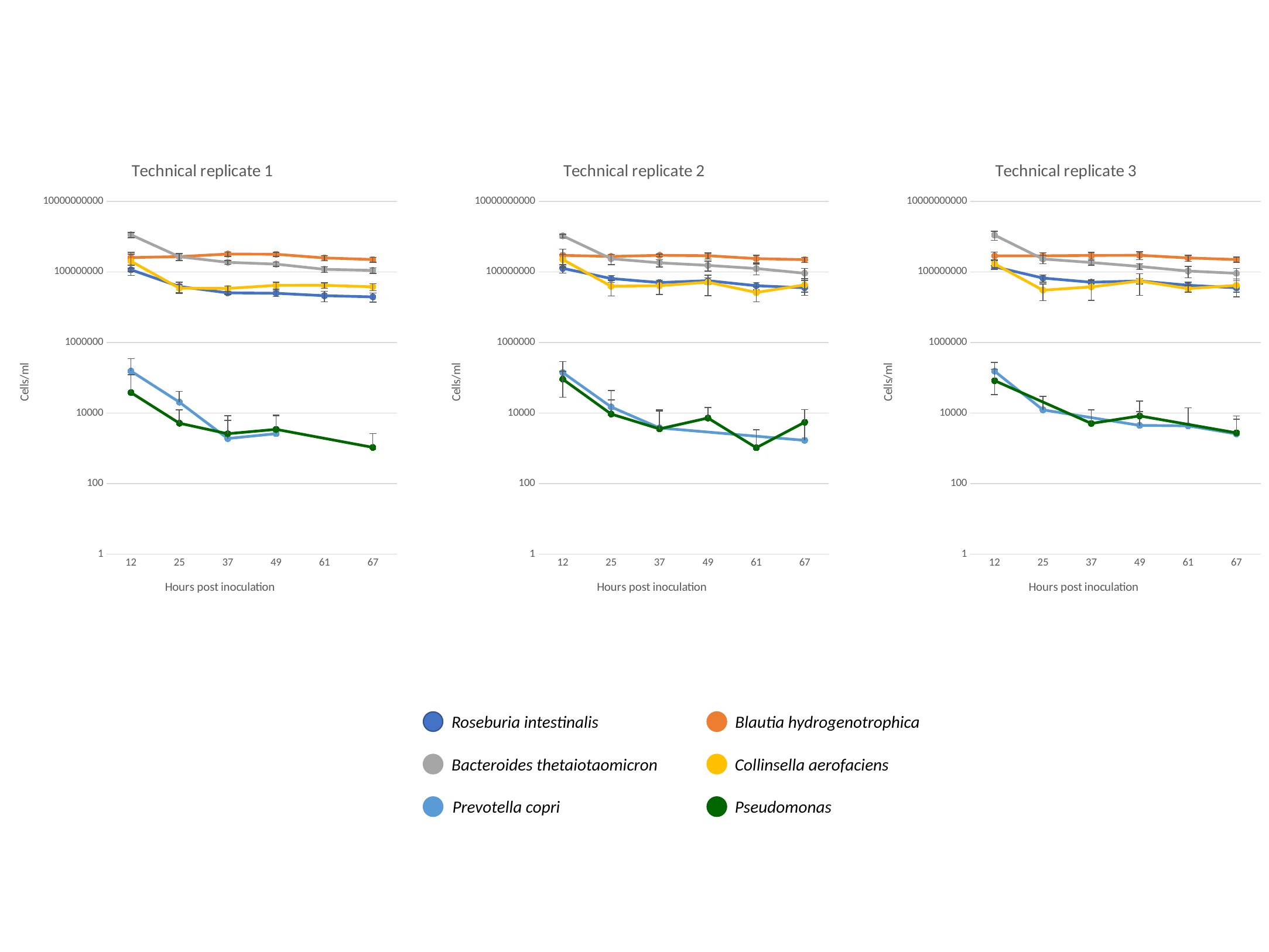

### Chart: Technical replicate 1
| Category | Roseburia intestinalis | Blautia hydrogenotrophica | Bacteroides thetaiotaomicron | Collinsella aerofaciens | Prevotella copri | Pseudomonas |
|---|---|---|---|---|---|---|
| 12 | 114559398.16314553 | 253874060.96642867 | 1120815669.9402304 | 199977655.46192193 | 154267.03891192118 | 37948.429360419505 |
| 25 | 38475186.71679466 | 270871701.07439715 | 268506867.1648348 | 34542248.832830064 | 20352.12448221553 | 5144.0866612673335 |
| 37 | 25433355.57140872 | 318991696.2871862 | 186278429.82118884 | 33882193.9054469 | 1897.20327589795 | 2593.8781601317664 |
| 49 | 24724219.977240413 | 315234252.8751169 | 165706675.86979267 | 41473642.11098812 | 2598.8900348705833 | 3443.6101602270614 |
| 61 | 21114165.762939345 | 247673492.21652636 | 117972793.59329319 | 41670048.427240886 | 0.0 | 0.0 |
| 67 | 19572967.925248686 | 223842284.1511723 | 109968576.59348698 | 37625774.77207978 | 0.0 | 1063.2246788593566 |
### Chart: Technical replicate 2
| Category | | | | | | |
|---|---|---|---|---|---|---|
| 12 | 125931260.05892392 | 293759119.7454773 | 1046659447.1025175 | 222837666.64417484 | 140620.51378787516 | 90885.9351179828 |
| 25 | 64612278.8105523 | 273127693.66308916 | 235513019.4808695 | 39144278.19611185 | 14863.60243509605 | 9366.246942191769 |
| 37 | 49723713.4274562 | 294081339.0251297 | 180207566.98799765 | 40570203.91580435 | 3790.2768826999168 | 3553.033395636967 |
| 49 | 56317081.794041775 | 287063362.33675617 | 152600111.15428647 | 51157053.25069568 | 0.0 | 7224.797553177584 |
| 61 | 40452116.79017464 | 237540220.58975852 | 124309864.01692738 | 26127257.800363306 | 0.0 | 1040.8027760315533 |
| 67 | 35384412.79055394 | 221679238.71589717 | 91290627.69324285 | 42649225.09931227 | 1683.5476304205333 | 5478.820030023303 |
### Chart: Technical replicate 3
| Category | | | | | | |
|---|---|---|---|---|---|---|
| 12 | 139353650.30384997 | 284497160.8096255 | 1096933783.3301294 | 168398123.45524183 | 153501.15230841417 | 82780.94884351257 |
| 25 | 66759063.036241435 | 284464392.0590133 | 230928139.12151885 | 30257615.677353185 | 12290.105873218583 | 0.0 |
| 37 | 50813119.391901515 | 292024498.4032583 | 184094494.77387133 | 37652987.47886559 | 0.0 | 5066.6187700042165 |
| 49 | 55136406.80112789 | 294991770.95448613 | 141899524.3564088 | 55104376.726569444 | 4467.3355541554 | 8287.1591866567 |
| 61 | 41750574.50778673 | 247620718.6159208 | 105540672.81018798 | 33514196.150684446 | 4337.915420007 | 0.0 |
| 67 | 35301898.7307693 | 223236745.18236455 | 91029846.12908113 | 41436878.32336444 | 2554.4100823716667 | 2743.891005024582 |Roseburia intestinalis
Blautia hydrogenotrophica
Collinsella aerofaciens
Bacteroides thetaiotaomicron
Prevotella copri
Pseudomonas

## Slide 5
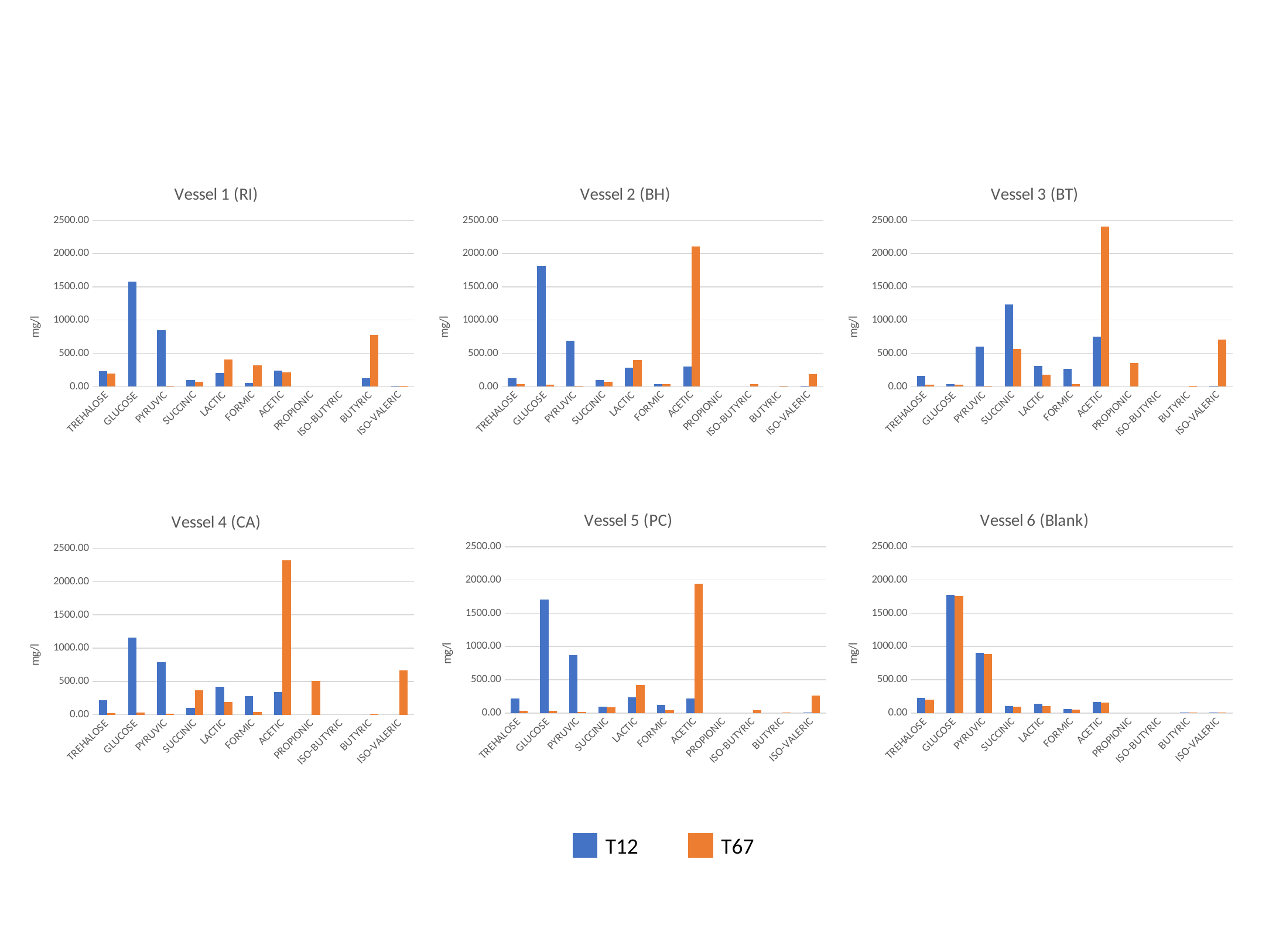

### Chart: Vessel 1 (RI)
| Category | T12 | T67 |
|---|---|---|
| TREHALOSE | 233.75 | 197.28 |
| GLUCOSE | 1575.15 | None |
| PYRUVIC | 845.04 | 12.41 |
| SUCCINIC | 96.88 | 75.39 |
| LACTIC | 202.96 | 404.66 |
| FORMIC | 58.14 | 317.34 |
| ACETIC | 243.26 | 214.4 |
| PROPIONIC | None | None |
| ISO-BUTYRIC | None | None |
| BUTYRIC | 128.49 | 778.75 |
| ISO-VALERIC | 9.04 | 6.38 |
### Chart: Vessel 2 (BH)
| Category | T12 | T67 |
|---|---|---|
| TREHALOSE | 126.97 | 35.6 |
| GLUCOSE | 1816.15 | 30.45 |
| PYRUVIC | 691.99 | 13.61 |
| SUCCINIC | 95.8 | 75.77 |
| LACTIC | 280.33 | 402.81 |
| FORMIC | 41.23 | 38.62 |
| ACETIC | 297.89 | 2111.09 |
| PROPIONIC | None | None |
| ISO-BUTYRIC | None | 40.79 |
| BUTYRIC | None | 7.48 |
| ISO-VALERIC | 10.58 | 183.61 |
### Chart: Vessel 3 (BT)
| Category | T12 | T67 |
|---|---|---|
| TREHALOSE | 162.29 | 26.77 |
| GLUCOSE | 38.19 | 30.03 |
| PYRUVIC | 604.86 | 14.52 |
| SUCCINIC | 1231.54 | 564.57 |
| LACTIC | 310.89 | 181.46 |
| FORMIC | 266.63 | 38.75 |
| ACETIC | 754.82 | 2406.41 |
| PROPIONIC | None | 354.75 |
| ISO-BUTYRIC | None | None |
| BUTYRIC | None | 1.32 |
| ISO-VALERIC | 13.21 | 702.8 |
### Chart: Vessel 5 (PC)
| Category | T12 | T67 |
|---|---|---|
| TREHALOSE | 221.73 | 29.2 |
| GLUCOSE | 1703.67 | 30.45 |
| PYRUVIC | 868.68 | 13.88 |
| SUCCINIC | 98.16 | 83.92 |
| LACTIC | 234.75 | 419.84 |
| FORMIC | 121.95 | 39.42 |
| ACETIC | 218.82 | 1940.32 |
| PROPIONIC | None | None |
| ISO-BUTYRIC | None | 42.26 |
| BUTYRIC | None | 4.89 |
| ISO-VALERIC | 8.3 | 260.58 |
### Chart: Vessel 6 (Blank)
| Category | T12 | T67 |
|---|---|---|
| TREHALOSE | 224.4 | 204.19 |
| GLUCOSE | 1778.48 | 1756.75 |
| PYRUVIC | 902.69 | 883.97 |
| SUCCINIC | 99.77 | 94.71 |
| LACTIC | 141.0 | 102.14 |
| FORMIC | 58.22 | 52.95 |
| ACETIC | 164.91 | 154.09 |
| PROPIONIC | None | None |
| ISO-BUTYRIC | None | None |
| BUTYRIC | 9.59 | 3.12 |
| ISO-VALERIC | 7.87 | 9.58 |
### Chart: Vessel 4 (CA)
| Category | T12 | T67 |
|---|---|---|
| TREHALOSE | 216.59 | 26.87 |
| GLUCOSE | 1162.65 | 31.1 |
| PYRUVIC | 787.6 | 16.53 |
| SUCCINIC | 99.35 | 364.76 |
| LACTIC | 422.67 | 191.42 |
| FORMIC | 273.52 | 38.04 |
| ACETIC | 343.06 | 2322.3 |
| PROPIONIC | None | 503.3 |
| ISO-BUTYRIC | None | None |
| BUTYRIC | None | 1.7 |
| ISO-VALERIC | None | 669.33 |T12
T67

## Slide 6
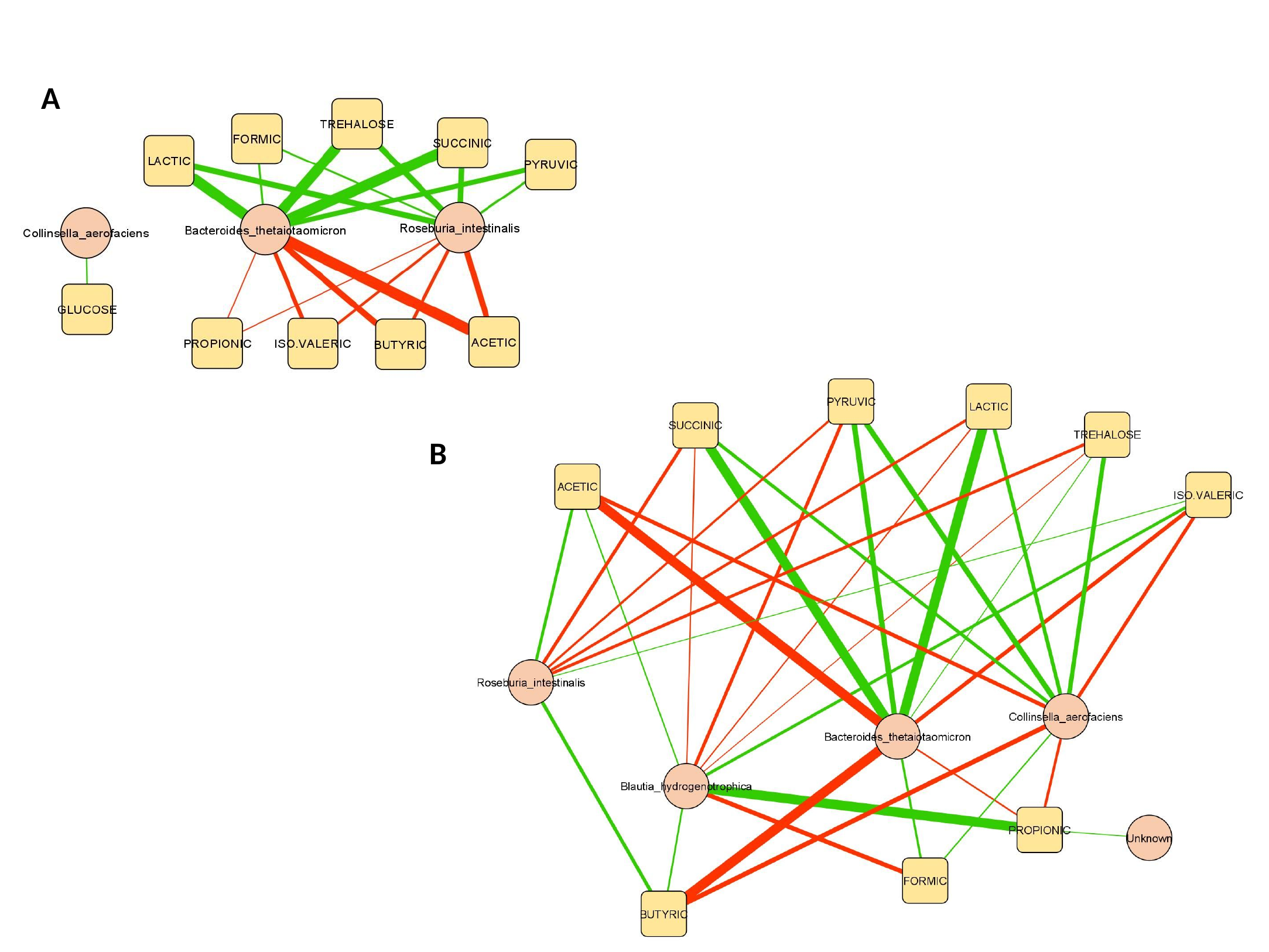

A
B

## Slide 7
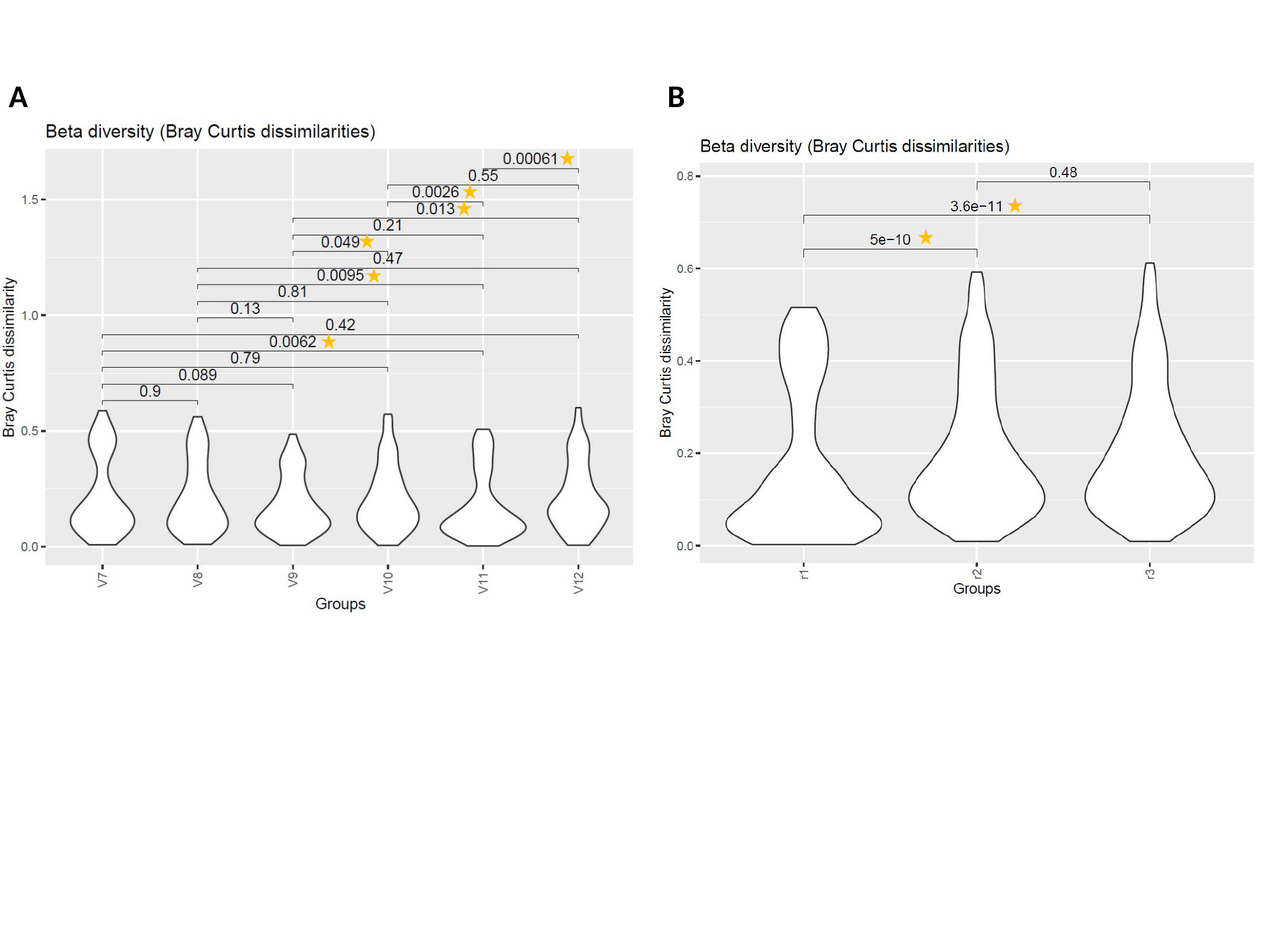

A
B

## Slide 8
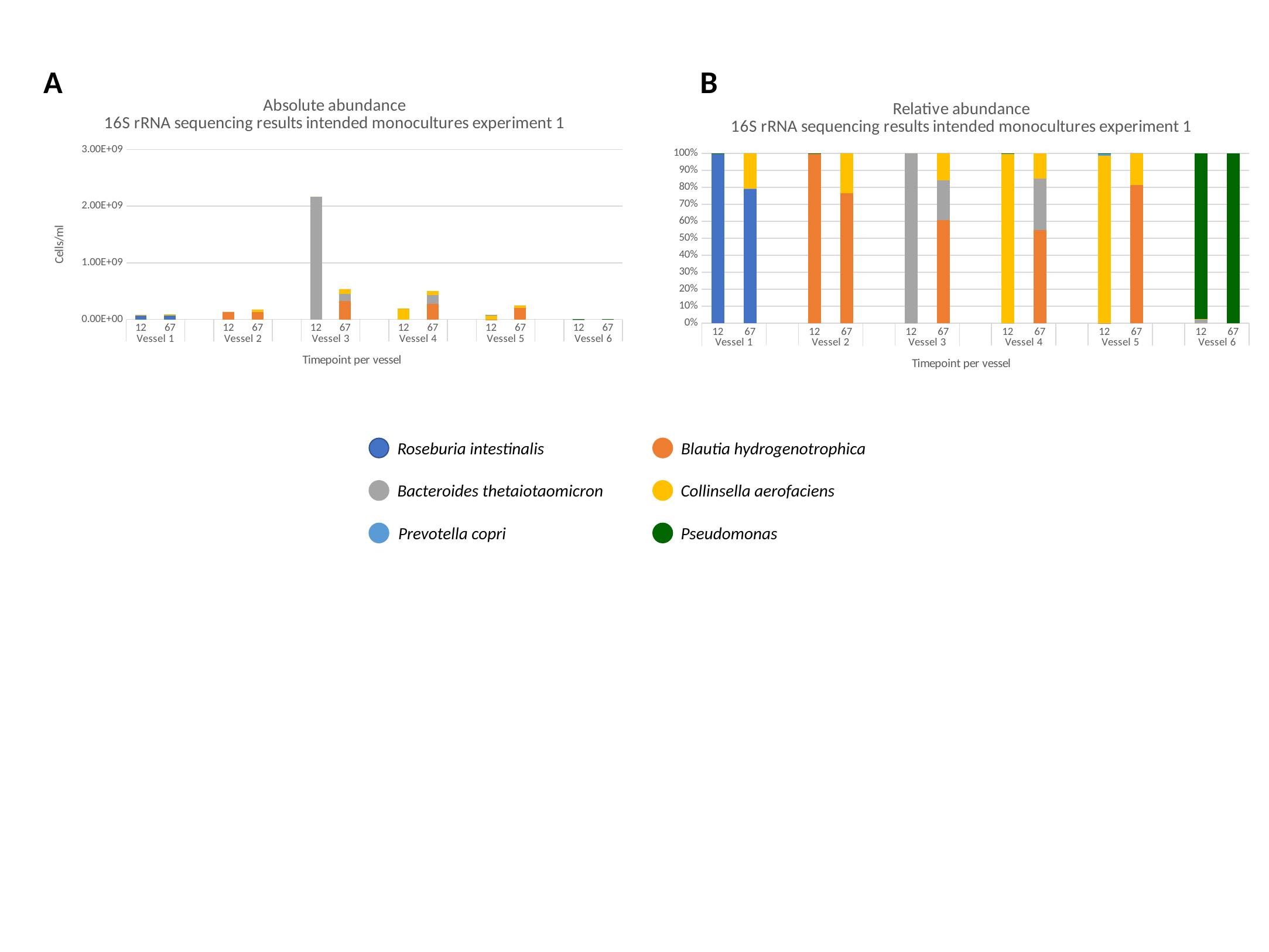

A
B
### Chart: Absolute abundance 16S rRNA sequencing results intended monocultures experiment 1
| Category | Roseburia intestinalis | Blautia hydrogenotrophica | Bacteroides thetaiotaomicron | Collinsella Aerofaciens | Prevotella copri | Pseudomonas |
|---|---|---|---|---|---|---|
| 12 | 82301724.5873154 | 0.0 | 0.0 | 361067.78736387 | 0.0 | 70207.6253207524 |
| 67 | 69882587.4524078 | 21357.2569029622 | 8542.90276118486 | 18362312.3388006 | 0.0 | 9200.04912742985 |
| | None | None | None | None | None | None |
| 12 | 0.0 | 131233720.343261 | 0.0 | 11427.5270239691 | 0.0 | 54852.1297150516 |
| 67 | 0.0 | 132042773.625478 | 0.0 | 40575636.4997567 | 0.0 | 3589.87476552329 |
| | None | None | None | None | None | None |
| 12 | 0.0 | 0.0 | 2167526809.23238 | 153190.767621659 | 0.0 | 0.0 |
| 67 | 0.0 | 325077558.811508 | 125309867.812208 | 84749076.3730105 | 0.0 | 6497.0032739384 |
| | None | None | None | None | None | None |
| 12 | 0.0 | 0.0 | 0.0 | 188433879.924953 | 0.0 | 106120.075046904 |
| 67 | 0.0 | 275928328.934616 | 151653777.201223 | 75069893.8641616 | 0.0 | 0.0 |
| | None | None | None | None | None | None |
| 12 | 0.0 | 64598.8842010911 | 0.0 | 82433344.1513283 | 1007742.593537021 | 94314.370933593 |
| 67 | 0.0 | 201956836.394576 | 0.0 | 46217895.0954495 | 23370.6993807896 | 11897.8105938565 |
| | None | None | None | None | None | None |
| 12 | 0.0 | 0.0 | 141201.716738197 | 28240.3433476395 | 0.0 | 6410557.93991416 |
| 67 | 0.0 | 0.0 | 0.0 | 0.0 | 0.0 | 2100000.0 |
### Chart: Relative abundance 16S rRNA sequencing results intended monocultures experiment 1
| Category | Roseburia intestinalis | Blautia hydrogenotrophica | Bacteroides thetaiotaomicron | Collinsella aerofaciens | Prevotella copri | Pseudomonas |
|---|---|---|---|---|---|---|
| 12 | 0.994787141615986 | 0.0 | 0.0 | 0.00436425353080234 | 0.0 | 0.000848604853211565 |
| 67 | 0.791565713520092 | 0.000241915374280302 | 9.67661497121207e-05 | 0.207991395256226 | 0.0 | 0.000104209699689976 |
| | None | None | None | None | None | None |
| 12 | 0.0 | 0.999495204442201 | 0.0 | 8.70337168619123e-05 | 0.0 | 0.000417761840937179 |
| 67 | 0.0 | 0.764924364365364 | 0.0 | 0.235054839474439 | 0.0 | 2.07961601969812e-05 |
| | None | None | None | None | None | None |
| 12 | 0.0 | 0.0 | 0.999929329620783 | 7.06703792172549e-05 | 0.0 | 0.0 |
| 67 | 0.0 | 0.60745923764584 | 0.234161463033634 | 0.158367158634254 | 0.0 | 1.2140686272526e-05 |
| | None | None | None | None | None | None |
| 12 | 0.0 | 0.0 | 0.0 | 0.999437148217636 | 0.0 | 0.000562851782363977 |
| 67 | 0.0 | 0.548945053306494 | 0.301707298889137 | 0.149347647804369 | 0.0 | 0.0 |
| | None | None | None | None | None | None |
| 12 | 0.0 | 0.000772713925850372 | 0.0 | 0.986044786499142 | 0.012054337243265802 | 0.00112816233174154 |
| 67 | 0.0 | 0.813653101787099 | 0.0 | 0.186204806798475 | 9.41569613665428e-05 | 4.79344530593309e-05 |
| | None | None | None | None | None | None |
| 12 | 0.0 | 0.0 | 0.0214592274678112 | 0.00429184549356223 | 0.0 | 0.974248927038627 |
| 67 | 0.0 | 0.0 | 0.0 | 0.0 | 0.0 | 1.0 |Roseburia intestinalis
Blautia hydrogenotrophica
Collinsella aerofaciens
Bacteroides thetaiotaomicron
Prevotella copri
Pseudomonas

## Slide 9
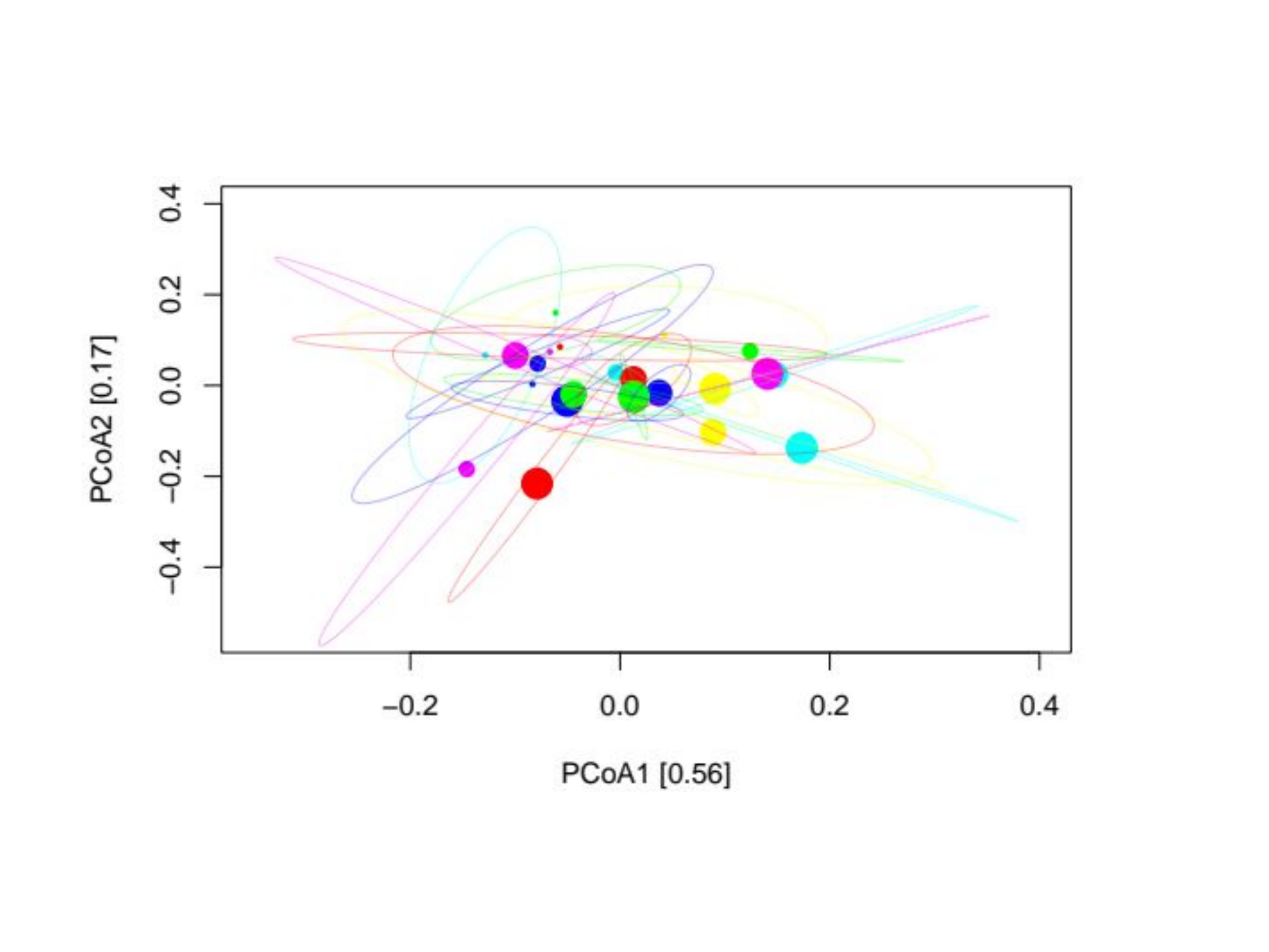

## Slide 10
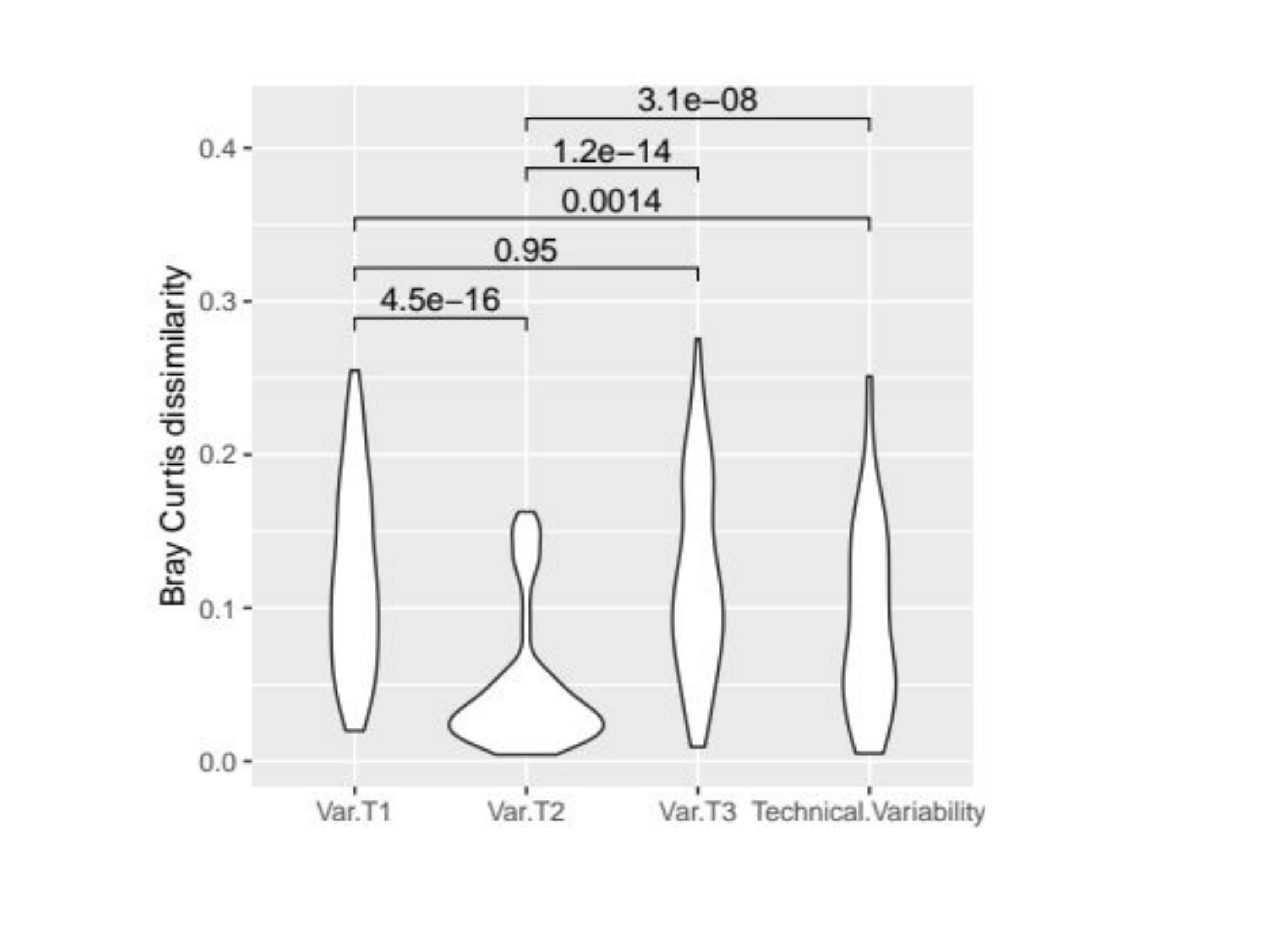

## Slide 11
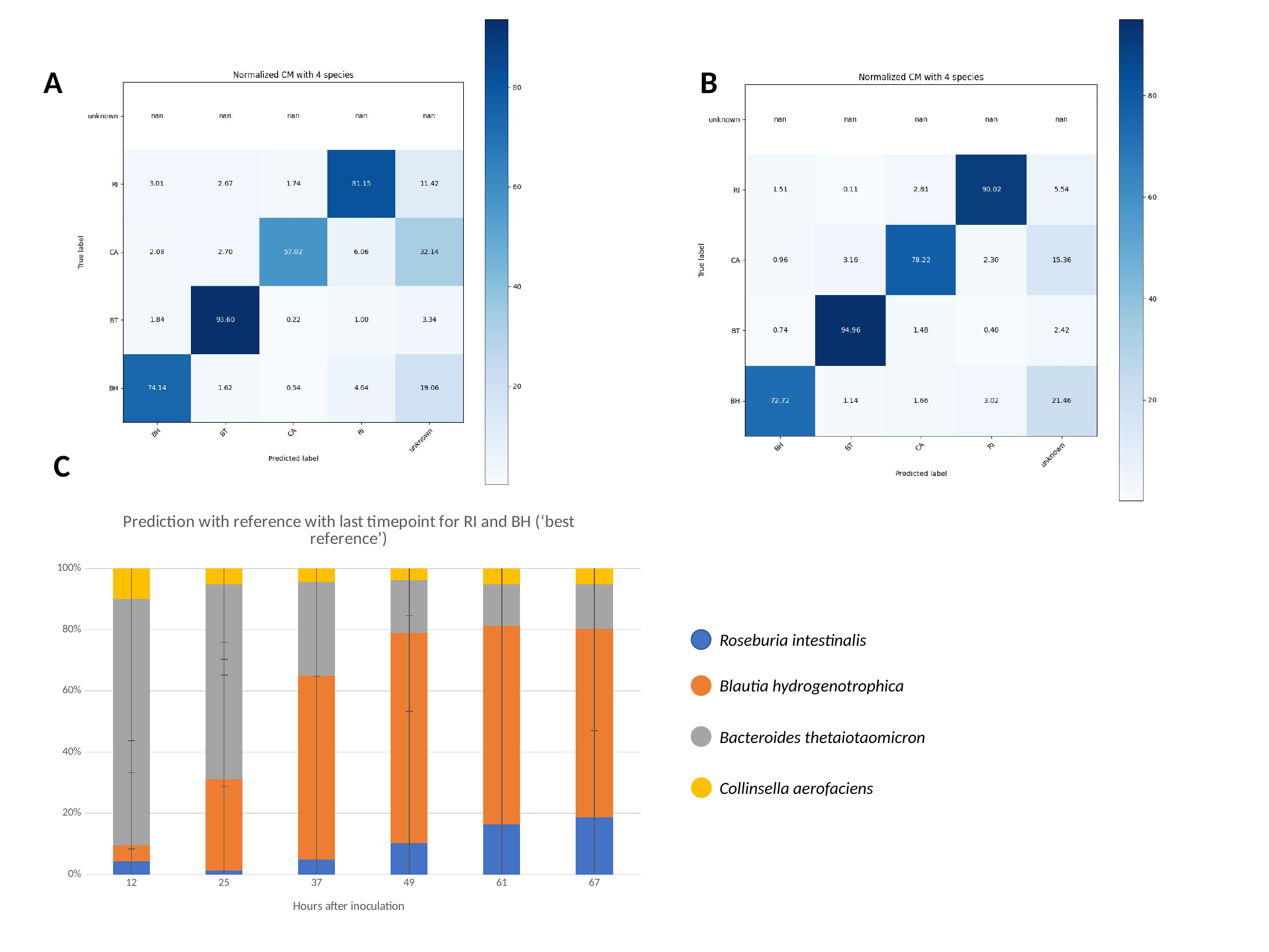

A
B
C
### Chart: Prediction with reference with last timepoint for RI and BH (‘best reference’)
| Category | Roseburia intestinalis | Blautia hydrogenotrophica | Bacteroides thetaiotaomicron | Collinsella aerofaciens |
|---|---|---|---|---|
| 12 | 4.2575647393919285 | 5.291511165376801 | 80.48762753060733 | 9.963296564623944 |
| 25 | 1.2827336999110766 | 29.811206612464137 | 63.65769809231923 | 5.24836159530556 |
| 37 | 4.822123549956679 | 60.18959416700519 | 30.581052067967672 | 4.407230215070456 |
| 49 | 10.263886140814 | 68.62552727420571 | 17.190587272937933 | 3.919999312042355 |
| 61 | 16.43895450272471 | 64.79355922680612 | 13.479371702219934 | 5.288114568249234 |
| 67 | 18.65572226705099 | 61.69464041417044 | 14.446560261104887 | 5.20307705767367 |Roseburia intestinalis
Blautia hydrogenotrophica
Bacteroides thetaiotaomicron
Collinsella aerofaciens

## Slide 12
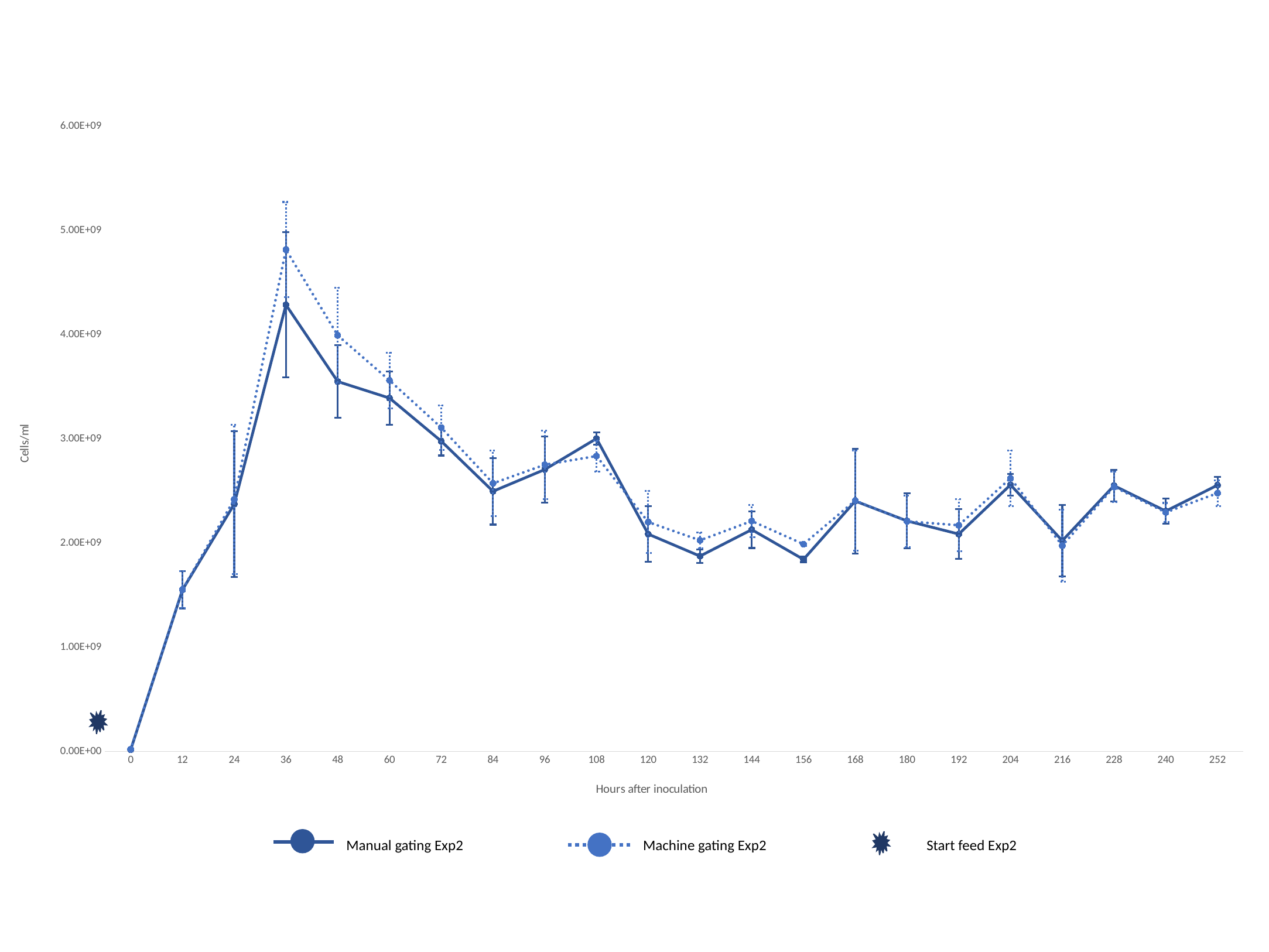

### Chart
| Category | Manual gating Exp2 | CellScanner gating Exp2 |
|---|---|---|
| 0 | 17293666.666666668 | 20807076.290078644 |
| 12 | 1551863666.6666667 | 1554743211.5733335 |
| 24 | 2373655000.0 | 2419107642.766667 |
| 36 | 4286575666.6666665 | 4816725594.533334 |
| 48 | 3550597666.6666665 | 3993465672.5733337 |
| 60 | 3392159666.6666665 | 3561459014.2333336 |
| 72 | 2978742333.3333335 | 3108724610.4733334 |
| 84 | 2497113666.6666665 | 2573539796.0533333 |
| 96 | 2705904000.0 | 2751890026.2000003 |
| 108 | 3003603000.0 | 2835662885.3733335 |
| 120 | 2086350666.6666667 | 2201935697.22 |
| 132 | 1874866333.3333333 | 2026147392.76 |
| 144 | 2129076000.0 | 2211609688.7000003 |
| 156 | 1843277000.0 | 1989858546.92 |
| 168 | 2403333000.0 | 2408148888.0866666 |
| 180 | 2212704333.3333335 | 2208751353.8266664 |
| 192 | 2086636000.0 | 2171336841.9933333 |
| 204 | 2559204000.0 | 2622189922.1 |
| 216 | 2022869000.0 | 1976209541.5 |
| 228 | 2551377000.0 | 2538456343.9266667 |
| 240 | 2307098000.0 | 2295134846.206667 |
| 252 | 2555802000.0 | 2479521653.08 |
Manual gating Exp2
Machine gating Exp2
Start feed Exp2

## Slide 13
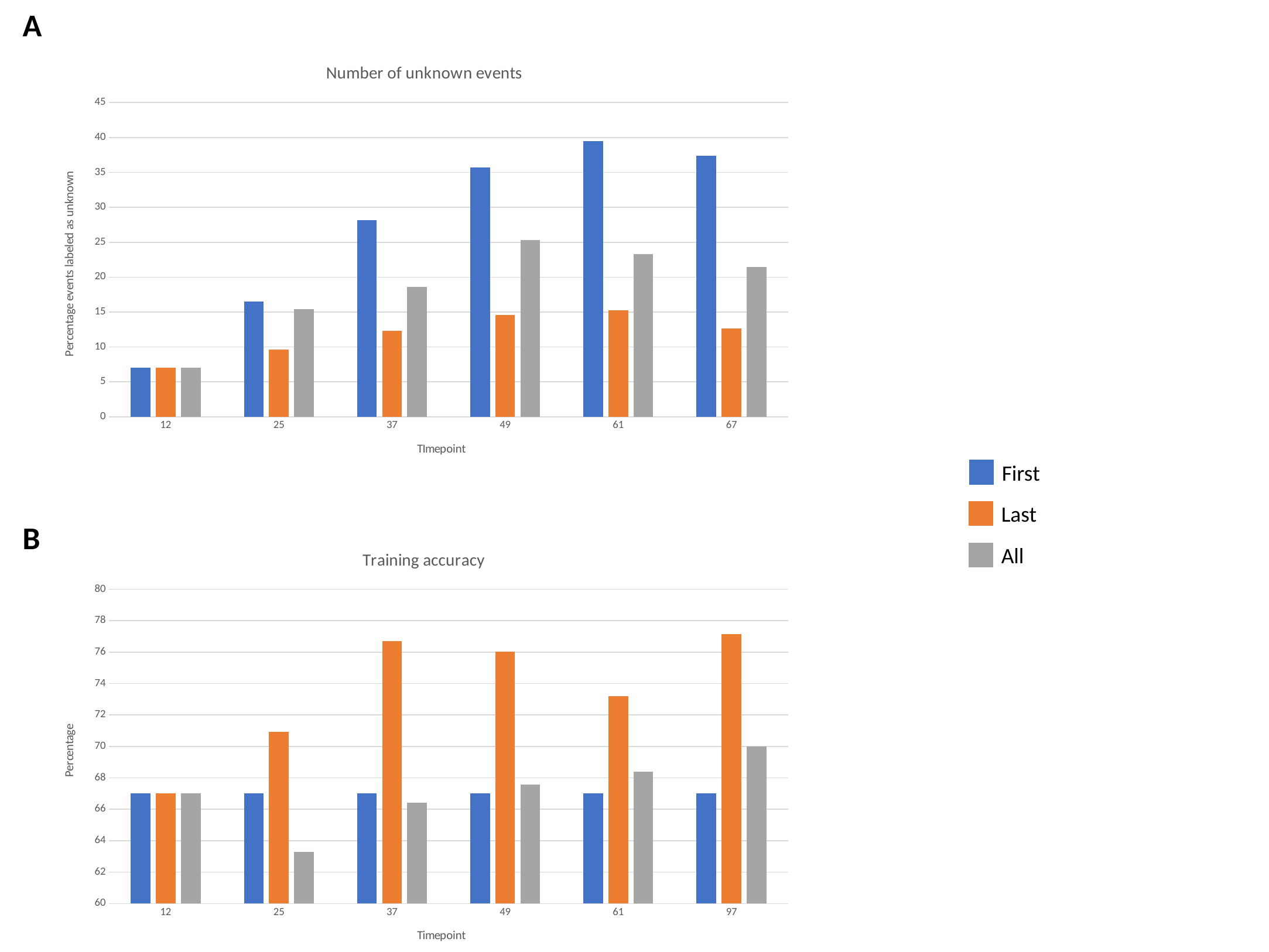

A
### Chart: Number of unknown events
| Category | | Last | All |
|---|---|---|---|
| 12 | 7.018029746476478 | 7.018029746476478 | 7.018029746476478 |
| 25 | 16.522189644673414 | 9.592776628518914 | 15.4085157980042 |
| 37 | 28.196409987421823 | 12.33056405918501 | 18.566711343993724 |
| 49 | 35.68796606739817 | 14.540693728486575 | 25.287719634164567 |
| 61 | 39.507632965147124 | 15.203308507663452 | 23.303595029340904 |
| 67 | 37.34529294385794 | 12.656823017301939 | 21.457393315840466 |First
Last
All
B
### Chart: Training accuracy
| Category | First | Last | All |
|---|---|---|---|
| 12 | 67.0 | 67.0 | 67.0 |
| 25 | 67.0 | 70.93 | 63.29 |
| 37 | 67.0 | 76.71 | 66.4 |
| 49 | 67.0 | 76.02 | 67.59 |
| 61 | 67.0 | 73.21 | 68.39 |
| 97 | 67.0 | 77.15 | 69.98 |
